# Childhood stunting and cognitive development: a meta-analysis

**DOI:** 10.7189/jogh.15.04257

**Published:** 2025-09-26

**Authors:** Vassilis Sideropoulos, Aisling Draper, Bernardita Munoz-Chereau, Lynn Ang, Julie Elizabeth Dockrell

**Affiliations:** 1 Department of Psychology and Human Development, UCL Institute of Education, University College London, London, UK; 2 Department for Learning and Leadership, UCL Institute of Education, University College London, London, UK

## Abstract

**Background:**

Childhood stunting is associated with delayed developmental trajectories. While the relationship between childhood stunting and cognitive development has been widely studied, the impact on children’s development requires further examination. We aimed to synthesise existing research studies to clarify the relationships between childhood stunting and cognitive development and sub-domains of cognition. Additionally, we sought to examine potential moderating factors influencing the relationship between childhood stunting and cognitive development, and to explore interventions targeted at improving cognitive development for stunted children.

**Methods:**

We systematically reviewed literature from 1990 to 2025 in 54 languages. We identified 12 191 studies, of which 35 studies met the inclusion criteria and were analysed. We employed random effect models to calculate pooled effect sizes and assessed heterogeneity using *I*^2^ statistics. We evaluated publication bias through Egger’s test.

**Results:**

Our initial model revealed no effects of childhood stunting on overall cognitive development. However, more refined domain-specific analyses showed that childhood stunting was associated with poorer sub-domains, specifically intelligence, executive function, visuo-spatial, cognition, and socio-emotional development in specific geographic regions. Secondary models indicated that the effects of childhood stunting were more pronounced when moderating factors such as demographics, socioeconomic, parent-related and health-related factors were controlled for, demonstrating the critical role of the impact of developmental context. Finally, the exclusive focus on nutritional interventions limited our ability to explore the effects of other intervention types on cognitive development in children who were stunted.

**Conclusions:**

Our findings highlight the need for further research to better understand these relationships and for the development of contextual interventions to draw robust conclusions and design targeted interventions. Future research should explore standardised culturally sensitive assessment tools, emphasising the necessity of accurate reporting, and the exploration of moderating effects across cognitive sub-domains.

The development of cognitive and socio-emotional skills impacts later employment, health, and well-being [1]. Addressing the link between early development and these long-term outcomes in low- and middle-income countries (LMICs) is of significant concern, as approximately 43% of children under the age of five are at risk of suboptimal development due to adverse conditions such as poverty and poor nutrition [2,3]. Nearly 80.8 million children aged 3–4 years in LMICs have low cognitive and socio-emotional development, with the highest prevalence in sub-Saharan Africa, South and East Asia, and the Pacific region [4].

Childhood stunting, a form of suboptimal growth due to malnutrition and repeated infections, is strongly linked to these developmental delays [4-6], and it is argued that its impact is particularly pronounced in early years (*e.g.* up to 60 months old) [7]. Childhood stunting not only hampers individual potential, such as employment and well-being, but also poses a global challenge [8], as these regions account for most of the world's population of children who are stunted.

Delayed developmental profiles for children who are stunted have been identified using assessments of cognitive, language, and motor development [4,9,10] with evident poorer academic attainment [11] and significant economic costs [12,13]. Childhood stunting and poverty are both predictive of poorer developmental trajectories. To date, research studies have indicated that childhood stunting accounts for a limited amount of variance in children’s performance on measures of cognition, social development, and academic attainment. Moreover, the effects are often only evident in children with early onset (<2 years) and persistent childhood stunting [14].

The biopsychosocial mechanisms postulated to drive the association between childhood stunting and developmental trajectories have varied. Some studies speak to the fact that early malnutrition impacts both growth and brain development [15], while others focus on learning opportunities, including a child’s reduced ability to engage with the environment due to poorer motor development and the lack of responsiveness by carers [14]. However, establishing causal relationships between child development and childhood stunting remains challenging, as a range of factors are associated with slower development in these populations. Factors include poverty, male gender, rural location, family characteristics, local environment, and the lack of cognitive stimulation [4,16,17]. Moreover, these factors are often confounded with other variables that impact development [18], making it difficult to establish whether childhood stunting is causally related to cognition or works as a proxy for social disadvantage and poorer learning environments [5].

A further challenge exists in the various ways in which infants’ and children’s cognitive development has been operationalised. For example, the impact of childhood stunting has been examined for oral language [19], gross and fine motor skills [20], problem solving [21], memory [22], executive function, and pre-academic attainment [23]. Some skills have been measured by tests standardised for the local population, while others are neither contextually nor culturally appropriate [22]. In other cases, non-standardised bespoke tasks have been developed for specific studies without consideration of the psychometric properties of the assessments. Some studies rely solely on parental reports, which may be impacted by the respondents’ knowledge and experience [24]. In sum, the diversity of measures used and the range of contextual factors that affect children’s development render it challenging to target areas for effective interventions to mitigate the impact of childhood stunting on children’s cognitive development. Researchers need to explore which interventions should be prioritised for which children, whether nutritional, social, or educational, in which contexts and whether the focus should be on the child, family, or locality [20,25,26]. Despite these challenges, numerous cross-sectional studies and two meta-analyses [9,10] have provided important evidence of a potential link between suboptimal linear growth and poorer child development.

Sudfeld and colleagues analysed data from 68 observational studies, which included objective assessments of children’s (<12 years) cognitive, motor, and socioemotional skills across 29 LMICs [9]. They identified a positive association between linear growth in the first two years of life and child development. However, impact was not differentiated by sub-domains/constructs of development, data were not analysed by LMICs status, and crucially, there was substantial variation which could not be attributed to linear growth alone. By contrast, Miller applied the Early Child Development Index, a tool that uses parental report to a ten-item questionnaire to capture developmental trajectories for literacy/numeracy, physical, social-emotional development, and learning, to track the development of children between the ages of 36–59 months across 15 LMICs [10,27]. Severe childhood stunting (height for age Z score (HAZ)<−3) was negatively associated with overall development, physical development, and learning across all the countries included. Results were more variable for literacy/numeracy, although when maternal education, sex of the child, wealth quintile, adult support for development, and number of books in the house were adjusted for, a significant impact of childhood stunting on literacy/numeracy in countries that reported higher levels of breastfeeding was identified. The data suggest a nuanced childhood effect stunting even when stunting is severe. However, detailed nuances in developmental trajectories may not have been identified as the results come from the ten-item Early Child Development Index, which uses parental report [28,29]. Parent-reported measures of development alone can be problematic as parents may report more socially desirable responses [30,31] and may not be aware of more subtle, mild to moderate delays, leading to a lack of precision in estimates [25,32]. Nonetheless, the range of countries included provides indicative evidence that context is essential for understanding the relationships between childhood stunting and child development.

Both previous meta-analyses point to a relationship between linear growth and domains of child development, but the skills impacted and the key moderating factors remain underspecified when robust objective measures of child development were used. Notably, neither of the previous reviews considered the region/country in which data were collected or the child’s age at the point of assessment. The age of assessment will influence the types of skills that can be assessed and potentially the sequelae of childhood stunting. As a result, local contexts will differ in the opportunities provided to children and their families. Given the importance of contextual factors on child development, this remains a limitation in our understanding of the relationships between childhood stunting and child development. Perkins and colleagues extended the examination of the relationships between child development and childhood stunting by providing a holistic and narrative review of the literature [5]. They suggested several aspects to be considered when examining the association between childhood stunting and children’s development if robust conclusions are to be obtained. These included addressing the heterogeneity in the methods used to assess development, unpacking covarying variables (including region/setting in which the data were collected), and capturing the differential impact of childhood stunting on singular aspects of cognitive development so that appropriate target domains for intervention can be identified and reliably tracked [26,33].

There are important considerations when using meta-analytic approaches to capture the effects of childhood stunting. Systematic/meta-analytic studies in the research literature often suffer from methodological flaws, as noted by Ioannidis, and if conducted improperly, they provide misleading conclusions [34]. In the context of childhood stunting, although data for different LMICs were available, these data were not analysed [9]. Failure to interrogate available data highlights the need for robust methods and transparency, particularly in areas like childhood development, where findings can directly influence interventions and policy. The current meta-analysis aims to address some of these limitations found in the literature by documenting which data were available for analysis, and where possible, utilising all data to ensure our conclusions are evidence-based and generalisable [6].

Building on previous seminal studies, we aimed to further explore the relationships between childhood stunting and cognition through a series of meta-analyses focussed on children who had experienced stunting under 24 months, the age at which the impact of stunting is most evident [7]. First, we examined relationships between childhood stunting and distinct domains of cognitive development to investigate whether these associations varied by global region and age of assessment. Second, we evaluated the influence of key confounding variables (*e.g.* age of assessment, demographic factors, health-related, and parent-related) when such data were available. Finally, we examined the current evidence purporting to demonstrate that interventions for stunted children impact their cognitive development. These aims were guided by the overarching question: To what extent, and under what conditions, does early childhood stunting influence cognitive development outcomes globally?

## METHODS

### Search strategy

We aimed to identify a comprehensive literature base across a broad range of geographical populations. To achieve this, we searched seven academic databases: PubMed, SCOPUS, Web of Science Core Collection, ERIC (ProQuest), British Education Index, Proquest Central (Proquest) and the International Bibliography of the Social Sciences (Proquest).

We undertook the searches up to 10 January 2025 for literature published from 1990 onwards. Despite the inclusion of all studies independent of language of publication, a search of studies published in 54 languages other than English (*i.e.* Afrikaans, Albanian, Arabic, Armenian, Azerbaijani, Bosnian, Bulgarian, Catalan, Chinese, Croatian, Czech, Danish, Dutch, Esperanto, Estonian, Finnish, French, Georgian, German, Greek, Modern, Hebrew, Hindi, Hungarian, Icelandic, Indonesian, Italian, Japanese, Kinyarwanda, Korean, Latvian, Lithuanian, Macedonian, Malay, Malayalam, Maori, Norwegian, Persian, Polish, Portuguese, Pushto, Romanian, Russian, Sanskrit, Scottish Gaelic, Serbian, Slovak, Slovenian, Spanish, Swedish, Thai, Turkish, Ukrainian, Vietnamese, and Welsh) identified no studies that met our criteria. The final data set included studies published in the English language. The searches combined a search term for childhood stunting with concepts relating to cognitive development sub-domains (Table S1 in the **Online Supplementary Document**), and the research team collaboratively developed the search strategy, informed by a preliminary searching exercise by the lead reviewer (JD). We also drew upon previous University College London’s Institute of Education and EPPI-Centre reviews to inform the search terms [35–37].

Inclusion criteria were: children between the ages of 0−24 months who were recorded as stunted from LMICs; any intervention targeting cognitive outcomes of a population who were stunted; cognitive development of children who were stunted in comparison to those who were not in the same locations; measures of cognitive development across the following sub-domains: academic skills, cognition, executive function, intelligence, memory, motor skills, oral language and visuo-spatial; published after 1990; and peer-reviewed publications.

Exclusion criteria were: studies not focussed on children who were stunted; children older than 24 months when childhood stunting was identified; stunting in populations within specific contexts (*e.g.* children with HIV); not an intervention targeting cognitive outcomes of a stunted population; not concerning stunted populations; not standardised measures of stunting such as HAZ or biomarkers; published before 1990; and study protocols.

Two researchers (AD and JD) independently screened titles and abstracts for inclusion using the online systematic review tool, EPPI Review. Once the decision criteria were agreed upon, they separately screened full-text articles for inclusion. Two authors (JD and BMC) provided adjudication on disagreement.

### Data extraction and quality assessment

**Figure 1:**
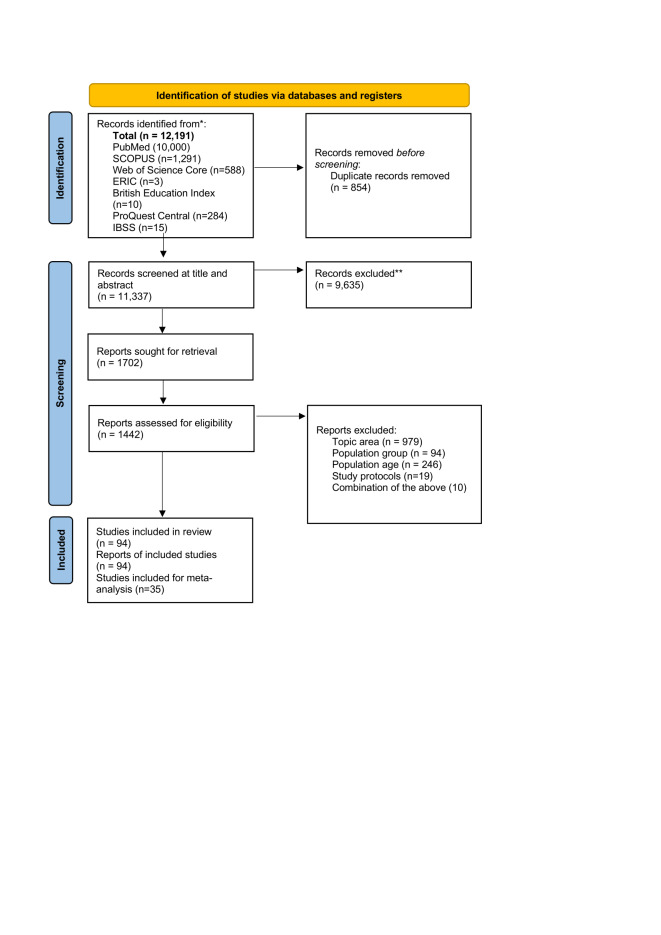
PRISMA flowchart

We imported the citations and abstracts returned from the academic database search into EndNote. We manually removed 854 duplicates (**Figure 1**). We imported the references into EPPI Reviewer for title and abstract screening.

We created a coding tool within EPPI-Reviewer to extract data, which included study design, sample size and characteristics, intervention/comparison, and findings. We also extracted cognitive domains, alongside the specific tests used to measure cognitive outcomes. Each member of the research team, who met to compare results and resolve disagreements by discussion and consensus, piloted the data extraction on a subsample of studies. One reviewer (AD) applied the tool to the rest of the studies.

We conducted a quality appraisal of included studies using a standardised checklist for assessing the quality of quantitative studies [38]. We conducted further risk of bias during meta-analysis by running sensitivity analyses throughout our models. We applied the quality appraisal tool in duplicate across 10% of the included studies, resolving any discrepancies through discussion and consensus. One reviewer (AD) quality appraised the remaining studies, and another reviewer (JD) checked them (**Table 1**).

**Table 1:** Characteristics of the included studies

Study, year, and country	Year, country	Region	Sample size, n	Study design	Cognitive domain
Acharya et al. [39]	2019, India	Asia	1194	Cohort comparison	Academic skills
Acharya et al. [40]	2023, Nepal	Asia	2870	Cohort comparison	Cognition
Ajayi et al. [41]	2020, South Africa	Africa	1386	Cohort comparison	Cognition, motor skills
Ayalew et al. [42]	2020, Ethiopia	Africa	505	Cohort comparison	Academic skills
Beckmann et al. [43]	2021, South Africa	Africa	1277	Cohort comparison	Academic skills, executive function
Brou et al. [44]	2023, Ivory Coast	Africa	3522	Cohort comparison	Academic skills
Casale et al. [45]	2014, South Africa	Africa	1258	Cohort comparison	Socio-emotional development, cognition
Chang et al. [46]	2010, Jamaica	North America	196	Cohort comparison	Motor skills
Chen et al. [47]	2021, China	Asia	1293	Cohort comparison	Cognition, socio-emotional development, memory
Duc [48]	2011, Vietnam	Asia	950	Cohort comparison	Oral language
Duc [49]	2016, Vietnam	Asia	1459	Cohort comparison	Cognition, socio-emotional development, academic skills
Ernawati et al. [50]	2020, Indonesia	Asia	150	Cohort comparison	Cognition
Gashu et al. [51]	2016, Ethiopia	Africa	541	Cohort comparison	Cognition, academic skills
Gerber et al. [52]	2021, South Africa	Africa	1277	Cohort comparison	Executive function, academic skills
Grantham-McGregor et al. [53]	1997, Jamaica	North America	206	Experimental	Cognition, motor skills, memory, academic skills
Honja et al. [54]	2021, Ethiopia	Africa	178	Cohort comparison	Cognition, memory
Kesari et al. [55]	2010, India	Asia	150	Cohort comparison	Executive function, oral language, cognition, memory, visuo-spatial, motor skills
Koshy et al. [56]	2022, India	Asia	203	Cohort comparison	Intelligence
Koshy et al. [57]	2022, India	Asia	205	Cohort comparison	Socio-emotional development
Li et al. [58]	2016, China	Asia	1744	Cohort comparison	Oral language, cognition, intelligence, academic skills
Lim et al. [59]	2023, Ghana	Africa	3801	Cohort comparison	Academic skills, socio-emotional development
Nguyen et al. [60]	2018, Vietnam	Asia	1458	Cohort comparison	Cognition, motor skills, oral language
Panigrahi et al. [61]	2018, Vietnam	Asia	256	Cohort comparison	Cognition
Primasari et al. [62]	2023, Malaysia	Asia	130	Cohort comparison	Oral language, motor skills, socio-emotional development
Ramel et al. [35]	2012, USA	North America	62	Cohort comparison	Cognition, motor skills, oral language
Ronaasen et al. [63]	2017, South Africa	Africa	105	Cohort comparison	Oral language, socio-emotional development, cognition
Sandjaja et al. [64]	2013, Indonesia, Malaysia, Thailand, Vietnam	Asia	6746	Cohort comparison	Intelligence
Sanou et al. [65]	2018, Burkina Faso	Africa	532	Cohort comparison	Memory, cognition
Selvam et al. [66]	2016, India	Asia	412	Cohort comparison	Oral language, socio-emotional development, motor skills
Sokolovic et al. [67]	2014, India	Asia	1040	Cohort comparison	Memory, cognition, oral language, intelligence
Tarleton et al. [68]	2006, Bangladesh	Asia	191	Cohort comparison	Intelligence, cognition
Wahyuningsih et al. [69]	2020, Indonesia	Asia	128	Cohort comparison	Socio-emotional development
Walker et al. [70]	2000, Jamaica	North America	196	Cohort comparison	Intelligence, oral language, executive function, memory, visuo-spatial, cognition
Warsito et al. [71]	2012, Indonesia	Asia	58	Cohort comparison	Cognition
Webb et al. [72]	2005, Indonesia	Asia	92	Cohort comparison	Cognition

### Meta-analysis approach

Studies that were pooled into the meta-analysis utilised several variables for reporting their findings. To compare them, we undertook data transformations, extracting effect sizes (Cohen’s *d*) and creating standard errors (SEs). Due to inconsistencies in reporting accurate statistical outcomes, we undertook further data transformations. To ensure consistency across the studies, we used an online effect size converter to transform odds ratios to Cohen’s *d* [73]. To convert comparison data to Cohen’s *d* effect size, we used the Campbell Collaboration Practical Meta-Analysis Effect Size Calculator [74]. For unstandardised beta coefficients (b), we transformed them to t-values (*t*) to be able to identify differences from zero between the dependent and independent variables by using the following equation: *t* = b / SE. Finally, for studies which reported effect sizes but did not provide SEs, we computed SEs through 95% confidence intervals (CIs) using the following equation: (upper bound − lower bound)/3.92 [75].

To investigate the direct effect of childhood stunting on cognitive development in children, we ran a primary model and performed sub-analyses across various cognitive domains, as identified in the literature. In all models, we included the age at which the assessment was carried out and the geographical region as covariates, both entered as dummy variables. Two researchers (AD and VS) initially coded cognitive domains, with JD reviewing and refining the coding, resulting in the following eight sub-domains: cognition, memory, visuo-spatial, intelligence, motor skills, academic achievement, socio-emotional development, and executive functioning. These sub-analyses enabled an exploration of the effects directly, gain tailored insights, offer targeted recommendations, and conduct sensitivity analyses.

Given the observed variability in effect sizes across studies, we expected high levels of heterogeneity. To address this, we employed random effects multilevel meta-analysis models, which account for between-study variation and are appropriate when studies differ in their populations, methods, and outcome measures. This approach allowed us to include a wide range of studies while acknowledging potential differences. While some *I*^2^ values were high, we proceeded with pooled estimates when theoretical rationale, model robustness, and consistency in direction of effects supported meaningful synthesis.

For assessing variables which moderated the effects of childhood stunting, we applied a similar approach. However, inconsistent reporting of results and data in the literature prevented the extraction of needed information to assess the effect of moderators on the relationship between childhood stunting and cognitive development. To address this, we introduced a dummy variable thematised into four categories: demographics, socioeconomic status, health-related information, and parental and household information, to capture the type of covariates authors included in their statistical model (Table S2 in the **Online Supplementary Document**). This approach enhanced our understanding of effects that were more likely to emerge. The dummy variable enabled us to compute combinations of those four themes, and study which combination was best explained by the data (*i.e.* D *vs.* S *vs.* D + S).

To facilitate comparisons across studies from different parts of the world, we grouped countries into broader regions (*e.g.* Asia, Africa, North America (Table S3 in the **Online Supplementary Document**). This approach was necessary due to data distribution: some regions were represented by only a few studies, while others were overrepresented. Grouping by region allowed us to maximise statistical power while preserving key geographical insights. In each analysis, we utilised a reference group selected from the most complete regional data available for the specific outcome being examined. For example, intelligence data were only available from North American and Asian studies, so North America was used as the reference group. In contrast, socio-emotional development data were only available from Asia and Africa, so Africa was used as the reference. While we recognise that the use of varying reference groups may also contribute to heterogeneity, we opted against a homogenised analytical approach to better reflect contextual diversity. We used this analysis for all sub-analyses. Given the different reference groups across measures, we examined each comparison separately. Similarly, given the heterogeneity in age of reporting and the varying ages at which cognition and stunting were assessed across studies, we binarised age into two categories: <60 months (*i.e.* five years) and >60 months. We reasoned that the age of five is a point when many children enter formal education and may influence developmental trajectories.

For our third research question, we did not analyse the impact of different interventions on cognitive development in children who were stunted because our search, despite using broad keywords, only led to the identification of nutritional interventions. Finding only one type of intervention made the comparison impossible and would have misrepresented the research focus. Finally, we assessed for publication bias with funnel plots, Egger’s test for meta-analysis and heterogeneity tests. Analyses were conducted in *R*, version 2023.06.0+421 (RCoreTeam, Vienna, Austria) using the metafor package [76].

## RESULTS

Descriptive analyses revealed that most studies were conducted in Asia (n = 20), followed by Africa (n = 11), whereas North America was less represented (n = 4). Out of the 35 studies, 28 relied on measures of children’s development (80%), six (17.1%) on parent-reported data [40,44,47,49,66,71], and one (2.9%) was unclear [63]. To provide an overview of the findings across all model outputs, we summarised the main results (**Table 2**).

**Table 2:** Summary of statistical models*

Domains	Overall effect	Age effects	Regional effects	Moderator effects
Overall cognitive development	No effect	No effect	No effect	No effect
Cognition	Suboptimal	No effect	No effect	No effect
Intelligence	Suboptimal	No effect	Yes, Asia	No effect
Executive function	Suboptimal	No effect	Yes, Asia	N/A†
Visuo-spatial skills	Suboptimal	No effect	Yes, Asia	N/A†
Socio-emotional development	Suboptimal	Yes, >60 mo	Yes, Asia	Demographics
Academic skills	No effect	No effect	No effect	No effect
Motor skills	No effect	No effect	N/A†	N/A†
Oral language	No effect	No effect	No effect	No effect
Memory	No effect	N/A†	No effect	N/A†

*To capture effects, adjustments were made for demographics, socioeconomic, health-related, parental, and household factors (if available).

†The model was not fully computed for the analysis/component due to missingness.

### Direct effect of overall cognitive development on childhood stunting

First, we conducted a multivariate meta-analysis of 20 studies with 152 *d* effect sizes obtained from a mean of 407.4474 participants (20 independent samples) to examine the association between age and region on cognitive development of children who were stunted. The model fit statistics indicated an acceptable fit, and the analysis revealed significant between-study variability (variance = 1.1638) as well as within-study variability (variance = 0.5011), suggesting heterogeneity in cognitive development outcomes for children who were stunted, both between and within studies.

However, the test of moderators did not show statistically significant effects on the cognitive development of children who were stunted (*Q* statistic for moderators (*Q_M_*)(3) = 1.0111; *P* = 0.7986). Further examination revealed that none of the individual predictors (*i.e.* age of assessment and region factors (Asia, Africa, North America)) were statistically significant (RQ1 main model in **Table 3**; see forest plot and model’s variance components in Figure S1 and Table S4 in the **Online Supplementary Document**, respectively).

**Table 3:** Coefficient summary from multilevel random effects main model

	Β (95% CI)	SE	Z	*P*-value
**RQ1 main model**				
Intercept	−1.0166 (−2.0792, 0.046)	0.5422	−1.8752	0.0608
Age	0.2351 (−0.535, 1.0052)	0.3929	0.5984	0.5496
Asia	0.3302 (−0.8132, 1.4736)	0.5834	0.5661	0.5714
North America	0.5529 (−1.0098, 2.1156)	0.7973	0.6934	0.488
**RQ2 main model**				
Intercept	0.5928 (−0.6534, 1.8389)	0.6358	0.9323	0.3512
Age	−0.627 (−1.6972, 0.4432)	0.5460	−1.1483	0.2509
Region – North America	0.0852 (−2.4819, 2.6523)	1.3098	0.0651	0.9481
Region – Asia	−0.9707 (−2.1599, 0.2186)	0.6068	−1.5997	0.1097
Combined factors present (D, P)	−1.2917 (−3.1219, 0.5384)	0.9338	−1.3833	0.1666
Combined factors present (D, S, H, P)	−1.6573 (−3.9748, 0.6602)	1.1824	−1.4016	0.1610
Combined factors present (D, S, H)	0.363 (−0.8506, 1.5765)	0.6192	0.5863	0.5577
Combined factors present (D, S)	−2.8421 (−6.0224, 0.3382 .)	1.6226	−1.7515	0.0799
Combined factors present (D)	0.9249 (−0.601, 2.4508)	0.7785	1.1880	0.2348
Combined factors present (H, P)	−0.5798 (−2.3413, 1.1816)	0.8987	−0.6452	0.5188
Combined factors present (H)	−0.2463 (−2.0457, 1.5531)	0.9181	−0.2683	0.7884
Combined factors present (S, H, P)	−0.1951 (−3.3928, 3.0025)	1.6315	−0.1196	0.9048

### Publication bias

We computed publication bias for the main model. The publication bias regression test was not significant (*P* = 0.775), suggesting no publication bias in the current meta-analysis. However, the heterogeneity test revealed substantial variability across studies in the study outcomes (χ^2^ (148) = 6000.585; *P* < 0.0001; tau squared (τ^2^) = 1.6648; *I*^2^ = 96%), indicating that approximately 96% of the total variability in effect sizes is due to true differences between studies rather than sampling error. This high heterogeneity also explains the asymmetry observed (Figure S2 in the **Online Supplementary Document**), indicating that it is more likely that studies with significant outcomes were published [77].

### Sub-analyses of the direct effect of cognitive development on childhood stunting

**Figure 2:**
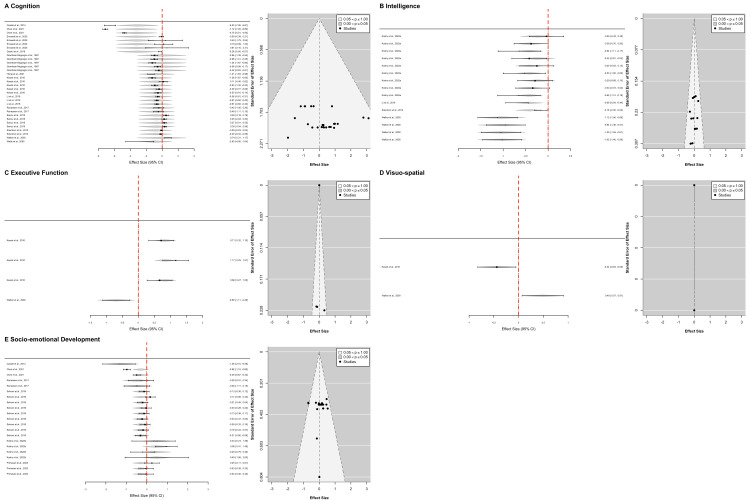
Forest plot with 95% CI accompanied by funnel plot for each sub-analysis.

To understand the unique effects of childhood stunting on domains of cognitive development, we computed a series of multivariate meta-analyses for each cognitive sub-domain identified in the studies. We present only significant model outcomes in the text, and the remaining are presented in the supplementary materials (Tables S5–8 in the **Online Supplementary Document**) along with coefficient summaries, variance components, heterogeneity tests, moderator tests, and publication bias. Forest plots with 95% CI and funnel plots were also computed (**Figure 2**).

### Cognition sub-domain

A multivariate meta-analysis model of 13 studies with 32 *d* effect sizes from the Africa, Asia, and North America regions with Africa used as the reference group, showed a significant negative intercept (*z = −*2.3640; *P* < 0.05; 95% CI = −5.6252, −0.5257), indicating that children who were stunted had suboptimal performance in tasks aiming to tap general cognition in the reference group (aged <60 months and studies conducted in Africa) compared to studies conducted in Asia and North America. A total between-study variance (τ^2^ = 4.68; *I*^2^ = 96.68) indicated that most of the observed variance was due to true differences across studies.

### Intelligence sub-domain

A multivariate meta-analysis model of four studies with 15 *d* effect sizes from the following regions: North America and Asia, with North America used as the reference group, revealed a significant negative intercept (z = −2.8173; *P* < 0.01; 95% CI = −1.4405, −0.2585), indicating that children who were stunted had suboptimal intelligence outcomes in the reference group (aged <60 months and studies conducted in North America) compared to studies that were conducted in Asia. Furthermore, a significant regional effect (z = 2.0218; *P* < 0.0.05; 95% CI = 0.0189, 1.2181) was observed for studies conducted in Asia. This suggests that the association between childhood stunting and intelligence outcomes may differ by regions, with the negative impact of childhood stunting on intelligence appearing less pronounced in Asia compared to North America. A total between-study variance (τ^2^ = 0.058; *I*^2^ = 58.49) indicated that more than half of the observed variance was due to true differences between studies.

### Executive function sub-domain

A multivariate meta-analysis model of two studies with four *d* effect sizes from North America and Asia, with North America used as the reference group, revealed a significant negative intercept (z = −2.3806; *P* < 0.05; 95% CI = −1.2523, −0.1213), indicating that children who were stunted had suboptimal executive function outcomes in the reference group (studies conducted in North America). A statistically significant regional effect was observed for studies conducted in Asia (z = 4.6027; *P* < 0.01; 95% CI = 0.8781, 2.1805). This suggests a regional differential effect in the association between childhood stunting and executive function. While childhood stunting was associated with lower executive scores overall, as indicated from the intercept, the magnitude of this association appears less pronounced in Asian studies compared to North American studies. A total between-study variance (τ^2^ = 0.002; *I*^2^ = 46.30) suggested that only 46% of the variability in effect sizes was due to real differences between studies, which potentially indicates greater consistency across findings in this sub-domain.

### Visuo-spatial sub-domain

A multivariate meta-analysis model of two studies with two *d* effect sizes from the North America and Asia regions, with North America used as the reference group, showed a significant intercept (z = 2.2891; *P* < 0.05; 95% CI = 0.0705, 0.9097), suggesting that children who were stunted had suboptimal performance in visuo-spatial tasks in the reference group (North American studies). However, a statistically significant regional effect was observed for studies conducted in Asia, specifically India (z = −3.1974; *P* < 0.01, 95% CI = −1.499, −0.3598). This indicates a complex relationship where the association between childhood stunting and visuo-spatial performance differs substantially between North American and Asian studies. Both τ^2^ and *I*^2^ of 0 showed that there was no detectable between-study heterogeneity, indicating that variation in effect sizes was entirely attributable to sampling error.

### Socio-emotional development sub-domain

A multivariate meta-analysis of six studies with 21 effect sizes from Asia and Africa, with Africa used as the reference group, revealed a significant intercept (z = −2.9838, *P* = 0.0028; 95% CI = −2.0341, −0.4212), indicating that children who were stunted had suboptimal socio-emotional development outcomes in the reference group (aged <60 months and studies conducted in Africa). A statistically significant relationship between age and socio-emotional development outcomes was observed (z = 2.0353; *P* = 0.0418; 95% CI = 0.0284, 1.5075), suggesting that older children who were stunted tended to score higher on socio-emotional development measures. Interestingly, a statistically significant regional effect was also observed in studies conducted in Asia (z = 2.2804; *P* = 0.0226, 95% CI = 0.1327, 1.7553). This suggests that the socio-emotional development outcomes differ between Asian and African studies, and the childhood stunting effect was less pronounced in Asian studies. Finally, between-study variance (τ^2^ = 0.116; *I*^2^ = 62.94) indicated moderate to substantial heterogeneity, suggesting that most of the proportion of the variability in effect sizes reflects true differences between studies for this sub-domain.

### Moderator effects of the relationship between cognitive development on childhood stunting

A multivariate meta-analysis of 22 studies with 125 *d* effect sizes obtained from a mean of 1282.856 participants (22 independent samples) was conducted to examine the association between childhood stunting, age at assessment, region, cognitive development and thematised moderators: demographics, socioeconomic information, health-related information, parental and household information. The model fit statistics indicated an acceptable fit, and the analysis revealed significant between-study variability (variance = 0.4677) as well as within-study variability (variance = 0.9032), suggesting heterogeneity in cognitive development outcomes for children who were stunted between and within studies.

The test of moderators – including age, region and thematised moderators – did not show statistically significant associations with cognitive development of children who were stunted (*Q_M_*(df = 11) = 17.0549; *P* = 0.1063). Further examination of the model results revealed that, from the individual predictors, age at assessment and region (Asia, Africa, North America), age at assessment, region (Asia, Africa, North America) and combined factors, the relationship between childhood stunting and cognitive development was not explained (Rq2 main model in **Table 3**; forest plot and model’s variance components in Figure S3 and Table S9 in **Online Supplementary Document**). Further examination is needed through sub-analysis.

### Publication bias

As before, publication bias was computed for the main model. The publication bias regression test was not significant (*P* = 0.837), suggesting no influence of publication bias in the current model. However, the assumption for the heterogeneity test did not confirm this finding (χ^2^(113) = 54828.0144; *P* < 0.001). By contrast, the τ^2^ = 1.36991 and *I*^2^ = 49.55067% implied moderate heterogeneity amongst studies, which suggested that around 50% of the total variation was due to real differences between studies rather than sampling error (Figure S4 in the **Online Supplementary Document**).

### Sub-analyses of the moderated effect of cognitive development on childhood stunting

**Figure 3:**
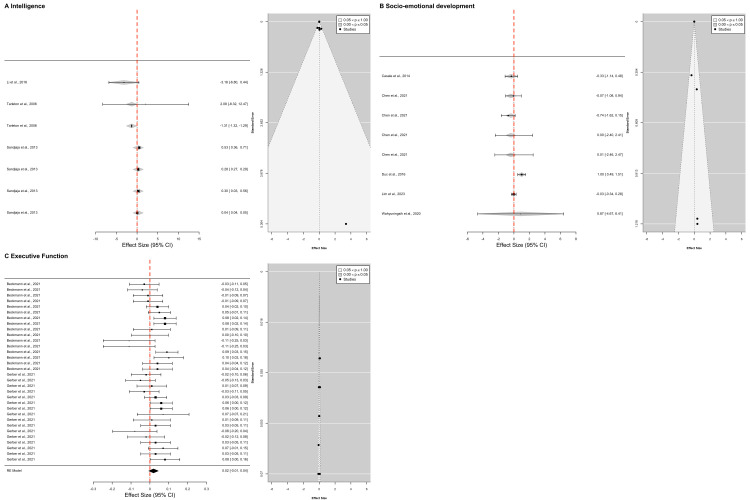
Forest plot with 95% CI accompanied by funnel plot for each sub-analysis.

We further computed a series of multivariate meta-analyses for each level of cognitive sub-domain to explore the unique effects of childhood stunting on these sub-domains. We outline below only significant model outcomes with the detailed outputs presented in the supplementary materials (Tables S10 and S13 in the **Online Supplementary Document**). We also computed forest plots with 95% CI and funnel plots (**Figure 3**).

### Intelligence sub-domain

A multivariate meta-analysis model of three studies with seven *d* effect sizes from the Asia region, showed a significant intercept (*z = −*2.0007; *P* < 0.001; 95% CI = −2.5777, −0.0265), suggesting that childhood stunting was associated with suboptimal performance in intelligence tasks in the reference group (aged >60 months in studies conducted in Asia and controlled for demographic and health factors in their models). No other factors in the model were significant. Finally, the model similarly showed that the total between-study variance was low (τ^2^ = 0.39), with minimal heterogeneity (*I*^2^ = 8.59%), suggesting that the observed variation in effect sizes may likely be due to sampling error and some true differences between studies.

### Socio-emotional development sub-domain

A multivariate meta-analysis model of five studies with eight d effect sizes from the Africa and Asia regions with Africa used as the reference group, showed that better socio-emotional development scores were observed for children who were stunted when studies accounted only for demographic variables in their models’ (*z* = 3.354; *P* = 0.0008, 95% CI = 0.5768, 2.1986) compared to when they accounted for parent/household, health-related factors and socio-economic information. This finding indicates that understanding socio-emotional development in children who were stunted requires a broader consideration of contextual factors such as parental/household characteristics, health-related factors and socio-economic characteristics. The model showed that the total between-study variance was low (*τ*^2^ = 0.001), with minimal heterogeneity (*I*^2^ = 0.0758%), indicating that most of the observed variation in effect sizes was likely due to sampling error rather than true differences between studies.

### Executive function

A multivariate meta-analysis model of two studies with thirty-two d effect sizes from the Africa region, showed a significant intercept (*z* = 3.1251; *P* = 0.0018; 95% CI = 0.0098, 0.0427), suggesting that childhood stunting was associated with suboptimal performance in executive-function tasks in studies conducted in Africa which adjusted for socio-demographic and health-related factors in their models – reflecting the residual effect of childhood stunting when controlling for contextual factors. The model showed that the total between-study variance was low (*τ*^2^ = 0.000), with small heterogeneity (*I*^2^ = 25.984%), indicating that the observed variation in effect sizes was likely due to sampling error rather than true differences between studies.

## DISCUSSION

Previous research has established that childhood stunting is associated with poorer overall cognitive development [9,10], although the causal drivers of these relationships are underspecified [5]. Given the current understanding of different trajectories for domains of development [78] and the impact of external factors on children’s cognitive development globally, we implemented a move away from a non-contextualised approach, allowing for a more nuanced exploration of the relevant data. We aimed to examine the effect of childhood stunting on the sub-domains of cognitive development (*i.e.* cognition, language, motor skill) [9,10]; to capture the moderators that contribute to the relationship between childhood stunting and cognitive development; and to evaluate evidence from impactful interventions. To achieve our three aims, we combined 277 effect sizes from 35 studies to run three separate random effects multilevel meta-regression models, with sub-analyses on eight cognitive development sub-domains. We identified several challenges in addressing our aims. Samples were not always well described, measures used varied, potential confounding variables were generally not reported, and there was significant variability in outcomes within and across studies.

In our main model addressing our first aim, there were no significant effects of childhood stunting on overall cognitive development. This may be because the effects of stunting differ across cognitive sub-domains and are shaped by other contextual factors, such as regions. Indeed, sub-domain analyses indicated that there were significant effects of childhood stunting for cognition, intelligence, executive function, visuo-spatial skills, and socio-emotional development, but not oral language, motor skills, memory, or academic attainment. The extent of these effects was influenced by geographical region. No detectable effects of childhood stunting were found on academic skills, motor skills, memory, and oral language, raising the question of how stunting impacts cognition and developmental sequelae.

Regional effects were influenced by the countries in which data were collected and the setting used as the reference point. Overall, the effects of stunting were more pronounced in Africa. By contrast, studies including data from Asia revealed less marked effects of childhood stunting [41,43,48,49,53,70,71,74,79–81]. Why the impact of childhood stunting is specifically evident in these sub-domains in these regions remains unclear and requires further examination. Diverse factors could explain these regional effects, including the broader socio-cultural context in which the children grow up and methodological features of the studies. Social determinants, including parental socioeconomic status, social and community context, health, access to healthcare, and quality, as well as parental social desirability, are potential confounds that only a few studies have controlled for when examining the impact of childhood stunting on children’s cognitive and socio-emotional development [30,31,47,58]. Inconsistencies across data sets and domains of cognition emphasise the need for a more rigorous and robust interrogation of methodological and analytic approaches [34,58]. This includes the number of studies underpinning the conclusions and whether models are adjusted for potential moderators of the association between childhood stunting and performance. Notably, the impact of childhood stunting on socio-emotional development diminished in the older children, a period when they would be attending school. This is another indication that wider cultural factors should be considered in the models.

Our secondary main model did not indicate that moderators were important exploratory variables when examined for overall cognitive development. However, sub-domain analyses revealed that demographics and health-related moderators explained better intelligence, socio-emotional and executive-function development in Asian and African regions. This model highlights the complex interaction between childhood stunting and the broader environmental and social disparities. Our findings are consistent with previous studies capturing the influence of similar moderators on cognitive and socio-emotional development in typical as well as atypical populations [82,83]. No moderating effects on academic skills, oral language, and motor skills were evident. Age of assessment was not found to be a significant factor in our secondary model and secondary sub-domain analyses, suggesting that the found impacts were evident independently of age of assessment. Finally, there were insufficient studies to compare the effects of different types of interventions on cognitive development in children who were stunted, with only three interventions with a nutritional focus, including some aspects of stimulation [46,53,70], and only one educational intervention [79] – making it impossible to compare effect sizes. Overall, the interventions identified ignored the broader context, focussing solely on nutritional and some limited stimulation aspects. The current approach in interventions aimed at childhood stunting and cognitive development fails to address the broader developmental context, factors which can support development and mitigate adversity. Future intervention studies must move beyond nutrition and consider contextually grounded educational interventions targeting development, ideally making comparisons across different localities [4,26,33,84].

Our main models indicated that there was no significant evidence of publication bias, as assessed by Egger’s test. However, the presence of substantial heterogeneity observed across all models and the asymmetry seen in some funnel plots suggest that potential publication bias cannot be ruled out. These indicators call for a more cautious interpretation of our findings, as they may reflect unreported or selectively published studies. Thus, while our statistical test did not confirm publication bias, its potential influence remains a concern given the variability in study quality, reporting practices, and outcome measures across included studies. Reproducibility and replicability are significant challenges in developmental and cognitive sciences [80,85], and capturing potential biases is essential in mitigating the ways forward in tackling the developmental profiles of stunted children. Similarly, publishing in developmental science is dominated by Western researchers. Although this gap has been shrinking, our analysis, which included studies from 1990 to 2025, exposed this bias, as a large proportion of the authors/co-authors in these studies were from Western countries [81], and we identified no studies published in languages other than English. Finally, heterogeneity in the samples from different countries restricted comparisons to regional differences, overlooking important country-specific variations.

Examining the measures used to assess cognitive development in the included studies also raised concerns. Culturally inappropriate, westernised, and standardised measures, along with a lack of reliable, valid, and appropriate measures for childhood development, were evident in many studies and the domains of development we have identified. English standardised assessments (*i.e.* Bayley and Peabody Vocabulary) often yield significant differences in cognitive performance across different cultures, making it challenging to draw conclusions [86,87] about how childhood stunting impacts development. Our previous work has highlighted the lack of culturally grounded, developmentally appropriate, and psychometrically sound assessments in early childhood research in LMICs [79]. Without proper tools, developmental outcomes can be misrepresented or obscured, limiting both the interpretability and applicability of findings. Moreover, parental health, well-being, education, child reading practices, and availability of resources, which have played a key role in cognitive performance in other studies, were considered as moderators in a minority of the included studies.

Studies also varied in their criteria for defining childhood stunting, with some studies explicitly stating the metric used for stunting classification (*i.e.* HAZ). In contrast, others created a binary category (*i.e.* stunted or not), without recourse to empirical data. This lack of standardisation poses significant limitations for meta-analytic synthesis, as not only is heterogeneity introduced in how the exposure variable is conceptualised and operationalised across studies, but additionally limits direct comparisons. This undermines the reliability of the pooled estimates, especially when assessing subtle regional and sub-domain differences. Similar reporting issues were identified in statistical outputs, psychometric properties, and sample details, limiting our ability to estimate effect sizes or to include key evidence from these sources in our analyses. This was particularly evident when intervention types were considered. Initially, we identified ten intervention studies, but only four of these provided sufficient statistical output details to allow for the computation of effect sizes.

In sum, the nature of the associations between childhood stunting and cognitive development remains underspecified and until this is clarified, interventions are unlikely to mitigate the damaging effects of overall poorer cognitive development. Our findings suggest that, for valid and impactful interpretations, an examination of the moderating effects of contextual factors in the relationship between childhood stunting and cognitive development, as well as sub-domain analyses, is required. Reliable, valid, and culturally appropriate measures – such as the cross-culturally comparable D-score – should be employed to improve consistency in assessing childhood cognitive development across LMICs [88]. Developing and validating such tools more widely across LMICs is essential to enhance the precision and comparability of future research. Our analyses also speak to the need for more consistency in the methods and approaches employed when studying cognitive development and childhood stunting – a common issue in developmental sciences. Finally, reporting of appropriate statistical outputs coherently and transparently – following open research practices and protocols – will ensure more reproducibility and replicability of these effect sizes [89]. These concerns are captured by Baird and colleagues and McCoy and colleagues, who discussed the methodological issues and analytical flexibility in intervention studies in childhood stunting and prompted researchers to utilise a holistic interventional approach with a focus on both socio-cultural and biological factors [4,84].

In addition to the limitations posed by study methods and available data, our categorisation of country types is problematic. Despite the novelty of moving away from ‘one-size-fits-all’ discourses and the practical relevance of this approach, we were restricted by the data available. This prevented the examination of likely differences within and between the country groupings and, as such, the potential impact of moderating variables. For example, while data from India and Indonesia were both classified as countries from Asia, they differ in child poverty rates, educational policies, and social policies, all of which could influence the impact of childhood stunting.

Despite this limitation, our meta-analysis highlights regionally specific effects of childhood stunting on cognitive development. Although childhood stunting may present differently across regions, the methodological challenges and high heterogeneity of the studies included in our analyses indicate that alternative interpretations should be considered. Future studies should employ reliable, valid, and culturally sensitive standardised cognitive assessments to improve comparability across studies, regions, and countries. This approach would help address the methodological issues identified in our analysis and reduce heterogeneity in results [87].

Moreover, future interventions aimed at tackling childhood stunting should be informed by holistic, context-sensitive approaches, considering not only the biological aspects of stunting – such as nutrition – but also the socio-cultural, economic, and environmental factors that shape its impact on cognitive development. Researchers should explore moderating effects, such as socioeconomic status, parental education, and health-related factors, and adopt standardised reporting guidelines to ensure consistency and robustness in future studies.

Comprehensive and multi-contextual understandings of childhood stunting and cognitive development will contribute to a clearer understanding of the relationship between early childhood stunting and cognitive development, ultimately leading to more impactful polices and interventions designed to mitigate the pervasive detrimental effects of childhood stunting.

## CONCLUSIONS

Our extensive meta-analysis demonstrates that the relationship between childhood stunting and cognitive development is more complex than many studies suggest. The context in which children grow and learn has often been overlooked, despite evidence from other fields showing its central role in shaping developmental trajectories. The diversity of studies in our review enabled subgroup analyses not only across regions and cognitive development sub-domains, but also across a range of contextual factors, offering further evidence that there are several moderators of this relationship. Researchers and policy makers need to consider which interventions should be prioritised for which children, whether nutritional, social, or educational, in which contexts, and whether the focus should be on the child, family, or locality if the global challenges of childhood stunting are to be addressed.

## Additional Material


Online Supplementary Document
