# Supplementary material for: Childhood stunting and cognitive development: a meta-analysis
Source: J Glob Health. 2025 Sep 26;15:04257. doi: 10.7189/jogh.15.04257 (PMC12467485; doi:10.7189/jogh.15.04257)
Supplement: Online Supplementary Document [file jogh-15-04257-s001.pdf]

Supplement to: Sideropoulos V, Draper A, Munoz Chereau B, Ang L, Dockrell JE. Childhood stunting and cognitive development: a meta-analysis. J Glob Health. 2025;15;04257.

Table S1. Search terms and searchers across databases.

**Name of the Database**	**SCOPUS**
**Platform**	
**Year Coverage**	**1990-present**
**Date of Search**	**31/05/2023**
**Total # of Results**	**2,033 BEFORE OR 1,290**
	**Article title, Abstract, Keywords**
**Refinements**	**Peer reviewed**
**Document Type (limit to)**	**Article / Review**
**Source type (limit to)**	**Journal**
	**saved**

	**Search Query**	**# of Results**
**1**	stunt* AND "cognitive development"	215
**2**	stunt* AND "motor development"	91
**3**	stunt* AND "child development"	940
**4**	stunt* AND "language development"	47
**5**	stunt* AND "cognitive ability"	33
**6**	stunt* AND "motor ability"	6
**7**	stunt* AND "language ability"	11
**8**	stunt* AND "child development" intervention	332
**9**	stunt* AND "early childhood development"	69
**10**	stunt* AND cognition	289
**11**	Combined with OR	1,290

**Name of the Database**	Web of Science core collection
**Platform**	
**Year Coverage**	01/01/1990 - 31/05/2023
**Date of Search**	**31/05/2023**
**Total # of Results**	**918 before OR --> 582**

	**Search Query**	**# of Results**
**1**	stunt* AND "cognitive development"	**211**
**2**	stunt* AND "motor development"	**80**
**3**	stunt* AND "child development"	**267**
**4**	stunt* AND "language development"	**23**

	TOPIC (title abstract key words)
Document type	article/ review article
	**saved**

**5**	stunt* AND "cognitive ability"	**18**
**6**	stunt* AND "motor ability"	0
**7**	stunt* AND "language ability"	2
**8**	stunt* AND "child development" intervention	120
**9**	stunt* AND "early childhood development"	66
**10**	stunt* AND cognition	131
**11**	combined with OR	582

**Name of the Database**	**ERIC**
**Platform**	Proquest
**Year Coverage**	**after 31/12/1989**
**Date of Search**	**31/05/2023**
**Total # of Results**	**10 BEFORE OR --> 3**
	**All abstract and summary text**
**Limit to**	**peer reviewed**
**document type**	**review / article / journal articles**

	**Search Query**	**# of Results**
**1**	stunt* AND "cognitive development"	1
**2**	stunt* AND "motor development"	0
**3**	stunt* AND "child development"	3
**4**	stunt* AND "language development"	1
**5**	stunt* AND "cognitive ability"	0
**6**	stunt* AND "motor ability"	0
**7**	stunt* AND "language ability"	0
**8**	stunt* AND "child development" intervention	0
**9**	stunt* AND "early childhood development"	0
**10**	stunt* AND cognition	0

	saved

11	combined with OR	3

**Name of the Database**	**British Education Index**
**Platform**	EBSCO
**Year Coverage**	**Jan 1990-May 2023**
**Date of Search**	**31/05/2023**
**Total # of Results**	**10 before OR --> 10**
	**scholarly/peer reviewed journals ticked**
**publication type**	**academic journal**
	**saved**

	**Search Query**	**# of Results**
1	stunt* AND "cognitive development"	3
2	stunt* AND "motor development"	0
3	stunt* AND "child development"	9
4	stunt* AND "language development"	1
5	stunt* AND "cognitive ability"	1
6	stunt* AND "motor ability"	1
7	stunt* AND "language ability"	0
8	stunt* AND "child development" intervention	0
9	stunt* AND "early childhood development"	1
10	stunt* AND cognition	2
11	combined with OR	10

**Name of the Database**	**Proquest Central**
**Platform**	Proquest
**Year Coverage**	**After 31/12/1989**
**Date of Search**	**31/05/2023**
**Total # of Results**	**372 BEFORE OR --> 263**
	**peer reviewed**
	**ALL abstract and summary text**
**document type**	**article**

	**Search Query**	**# of Results**
1	stunt* AND "cognitive development"	91
2	stunt* AND "motor development"	36
3	stunt* AND "child development"	98
4	stunt* AND "language development"	7
5	stunt* AND "cognitive ability"	8
6	stunt* AND "motor ability"	1
7	stunt* AND "language ability"	1
8	stunt* AND "child development" intervention	43
9	stunt* AND "early childhood development"	36
10	stunt* AND cognition	51

	saved

11	combined with OR	264

**Name of the Database**	**IBSS**
**Platform**	Proquest
**Year Coverage**	**After 31/12/1989**
**Date of Search**	**31/05/2023**
**Total # of Results**	**22 before OR -->**
	all abstract and summary text
	**Peer reviewed**
**document type**	**article**
	**Saved**

	**Search Query**	**# of Results**
**1**	stunt* AND "cognitive development"	4
**2**	stunt* AND "motor development"	1
**3**	stunt* AND "child development"	8
**4**	stunt* AND "language development"	0
**5**	stunt* AND "cognitive ability"	0
**6**	stunt* AND "motor ability"	0
**7**	stunt* AND "language ability"	0
**8**	stunt* AND "child development" intervention	2
**9**	stunt* AND "early childhood development"	1
**10**	stunt* AND cognition	6
**11**	combined with OR	15

**Name of the Database**	**PubMed**
**Platform**	**PubMed**
**Year Coverage**	**After 31/12/1989**
**Date of Search**	**31/05/2023**
**Total # of Results**	**1953 before OR --> 10,000**
	all abstract and summary text
	**Peer reviewed**
**document type**	**article**
	**Saved**

	**Search Query**	**# of Results**
**1**	stunt* AND "cognitive development"	132
**2**	stunt* AND "motor development"	43
**3**	stunt* AND "child development"	734
**4**	stunt* AND "language development"	25
**5**	stunt* AND "cognitive ability"	13
**6**	stunt* AND "motor ability"	344
**7**	stunt* AND "language ability"	287
**8**	stunt* AND "child development" intervention	159
**9**	stunt* AND "early childhood development"	71
**10**	stunt* AND cognition	145
**11**	combined with OR	10,000

Table S2. Thematised dummy variables.

Demographics	Sociodemographic	Health Nutrition	Parenting Home
Age	Household income	Height-for-age z-score (HAZ)	Maternal marital status
Child age	Scheduled caste	Weight-for-age z-score (WAZ)	Maternal education status
Girl	Scheduled tribe	Body Mass Index (BMI)	Maternal occupation
Sex	Wealth index	Z-score of Body Mass Index (zBMI)	Mother's PPVT (Peabody Picture Vocabulary Test)
Birth order	Asset Index	Haemoglobin level	Number of family living together
Female	Household Socioeconomic status (SES)	Stunted	Residents
Maternal age at childbirth	Father's education	Thinness	Caregiver plays for less than an hour
Mother's years of schooling	Mother's education	Underweight	Caregiver plays for more than an hour
Number of older half/siblings	English medium school	Birthweight	Caregiver teaches child
Both biological parents in household	No preschool education	Length in preterm	Father figure plays once a week
Area of residence	School books	Any chronic disease	Father figure plays 2-4 times a week
Region	Educational level of parents	Congenital or perinatal disorder	Father figure plays every day
County		HIV (human immunodeficiency virus) positive	Stimulation factors
Child school level		HIV (human immunodeficiency virus) unknown	Regular study
Presence of parental disability		STH (soil-transmitted helminth) infection	Child likes school environment
		Anaemia	Reading books
		Morbidity	Telling stories to child

Systemic inflammation
Gut inflammation
Enteropathogen load
Food insecurity
Worried about not having sufficient food
Dietary diversity
HH iodized salt use
Deworming
Breakfast before school
Lunch before school
Special foods
Grip strength
Cardiorespiratory fitness
Birth
Breastfeeding

Table S3. Country Classification.

Region	Country
Asia	India, China, Indonesia, Nepal, Vietnam, Bangladesh, Malaysia Thailand
Africa	Ethiopia, South Africa, Burkina Faso, Ghana, Ivory Coast
North America	Jamaica, USA

Main analysis on the direct effect of cognitive development in childhood stunting (3.1).

Table S4. Variance Components

	B	sqrt	N levels	Fixed	factor
Test for Residual Heterogeneity	$Q_E(148) = 6000.5847$, $p<.0001$				
sigma^2.1	1.1638	1.0788	20	No	Study ID
sigma^2.2	0.5011	0.7079	152	No	Effect Size

Figure S1. Forest plot of the 152 effect sizes along with their 95% confidence intervals (*Main analysis on the direct effect of cognitive development in childhood stunting (3.1)*).

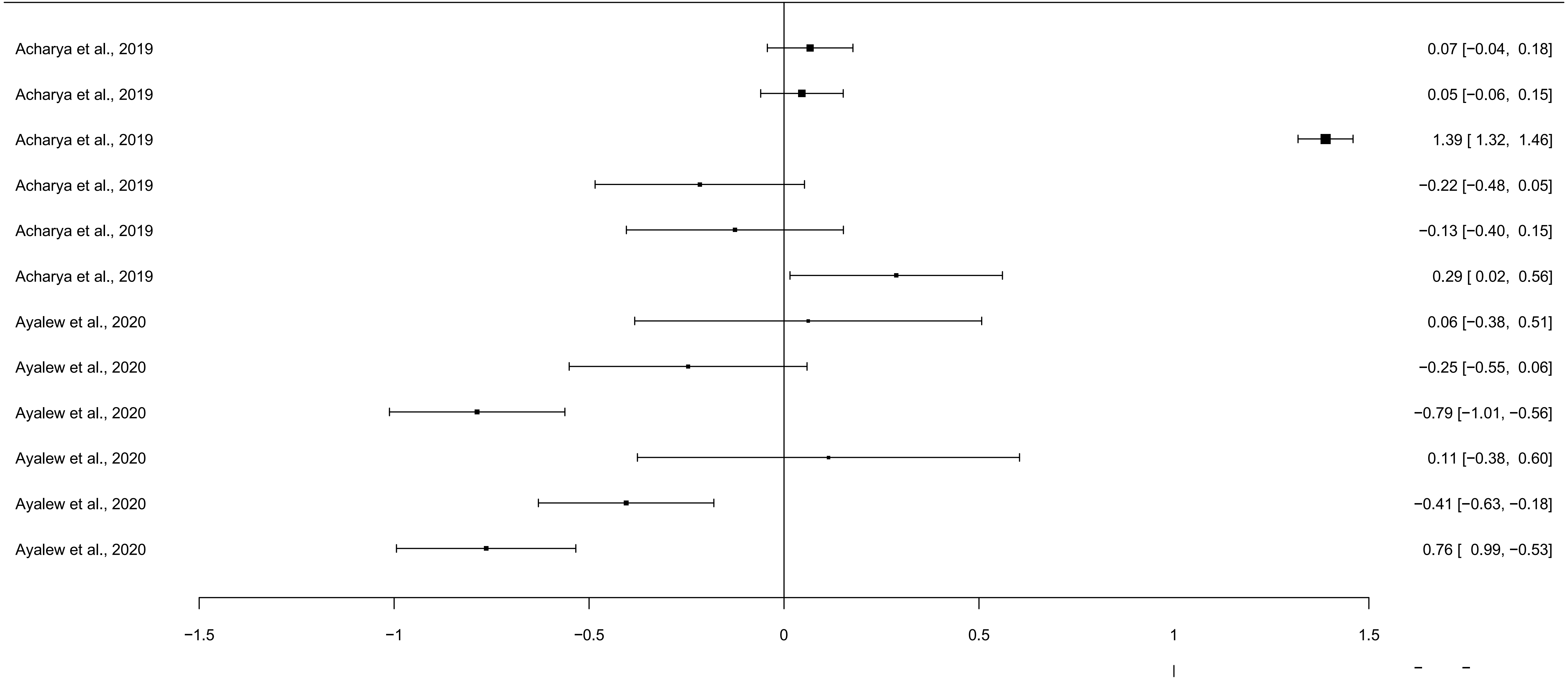

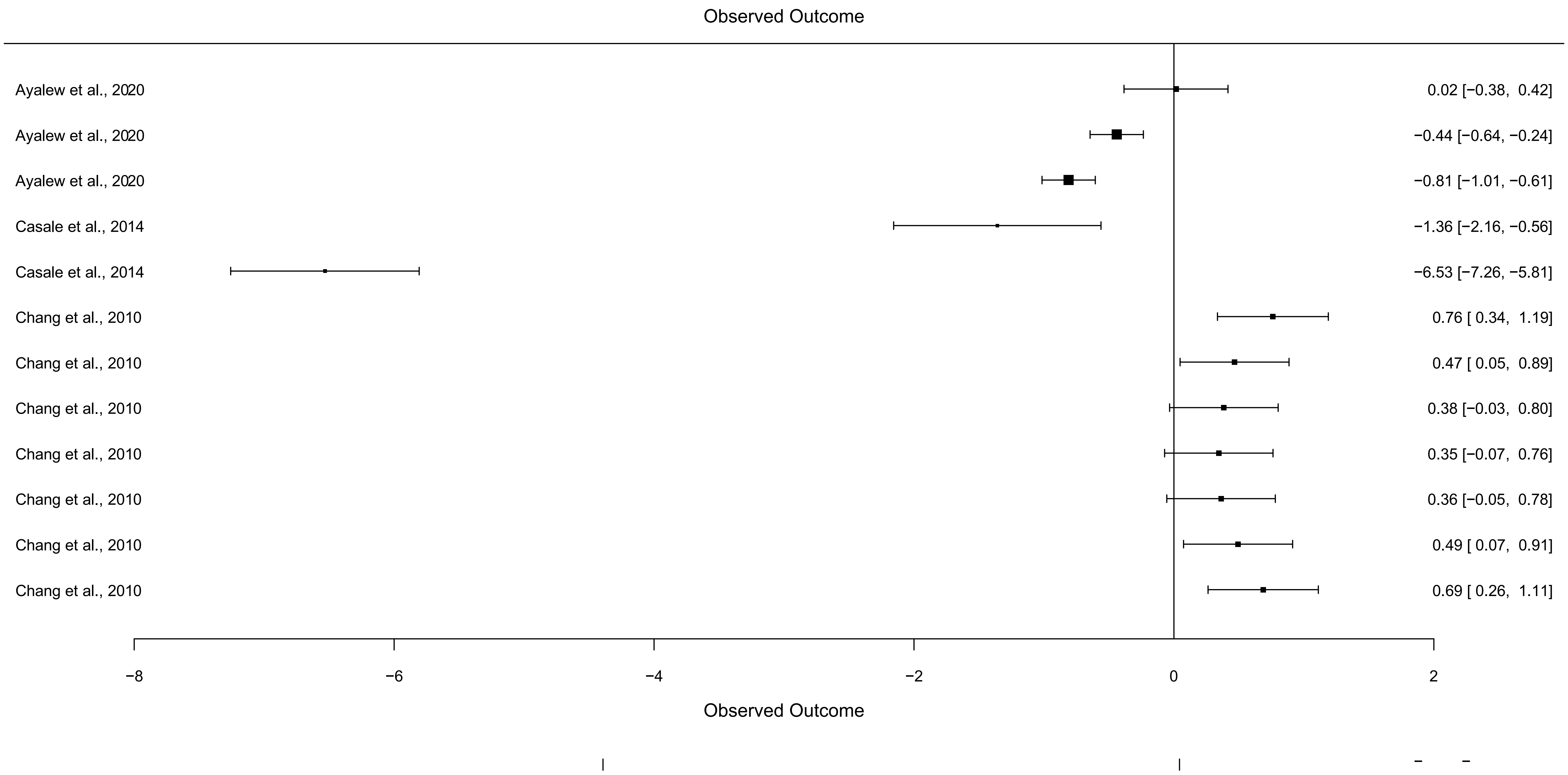

Observed Outcome
Ayalew et al., 2020
Ayalew et al., 2020
Ayalew et al., 2020
Casale et al., 2014
Casale et al., 2014
Chang et al., 2010
Chang et al., 2010
Chang et al., 2010
Chang et al., 2010
Chang et al., 2010
Chang et al., 2010
Chang et al., 2010
0.02 [−0.38, 0.42]
−0.44 [−0.64, −0.24]
−0.81 [−1.01, −0.61]
−1.36 [−2.16, −0.56]
−6.53 [−7.26, −5.81]
0.76 [0.34, 1.19]
0.47 [0.05, 0.89]
0.38 [−0.03, 0.80]
0.35 [−0.07, 0.76]
0.36 [−0.05, 0.78]
0.49 [0.07, 0.91]
0.69 [0.26, 1.11]
−8
−6
−4
−2
0
2
Observed Outcome

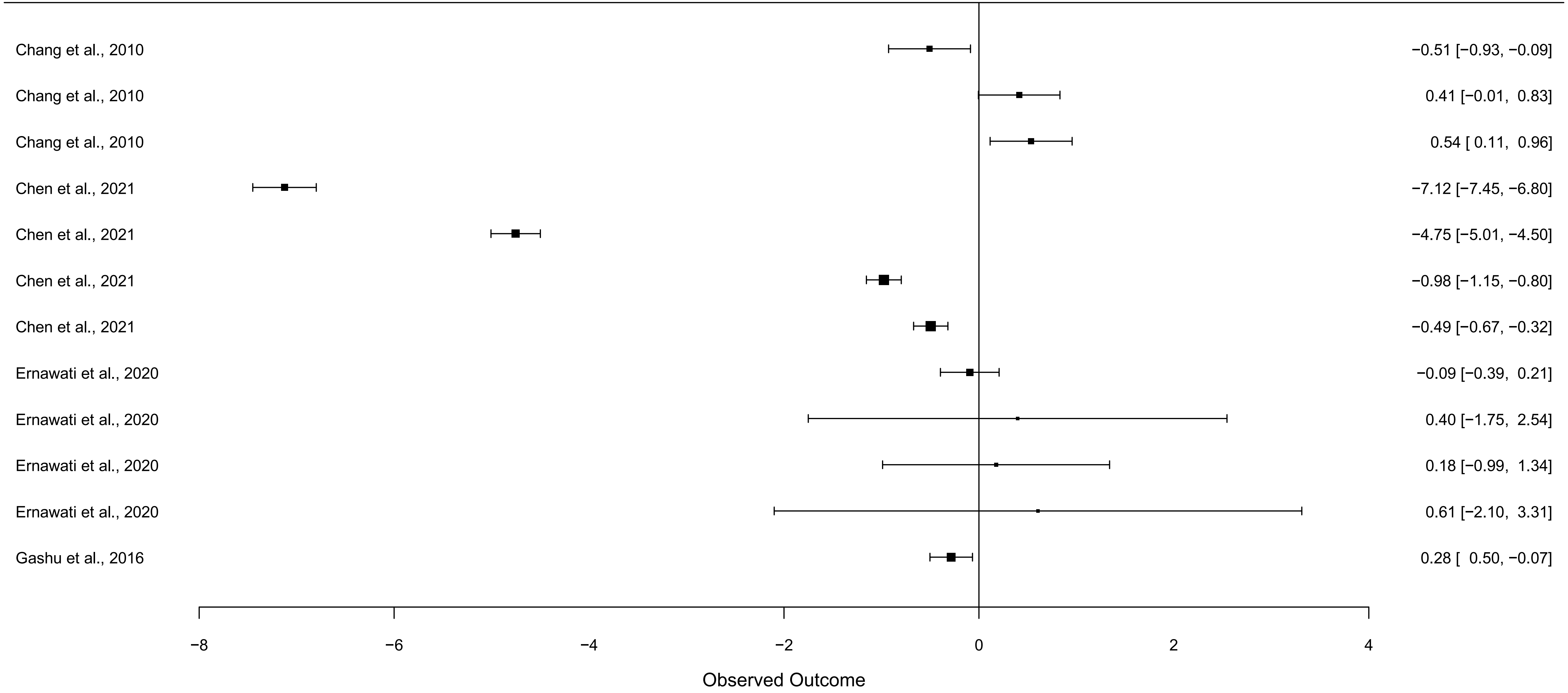

Chang et al., 2010
Chang et al., 2010
Chang et al., 2010
Chen et al., 2021
Chen et al., 2021
Chen et al., 2021
Chen et al., 2021
Ernawati et al., 2020
Ernawati et al., 2020
Ernawati et al., 2020
Ernawati et al., 2020
Gashu et al., 2016
−0.51 [−0.93, −0.09]
0.41 [−0.01, 0.83]
0.54 [0.11, 0.96]
−7.12 [−7.45, −6.80]
−4.75 [−5.01, −4.50]
−0.98 [−1.15, −0.80]
−0.49 [−0.67, −0.32]
−0.09 [−0.39, 0.21]
0.40 [−1.75, 2.54]
0.18 [−0.99, 1.34]
0.61 [−2.10, 3.31]
0.28 [0.50, −0.07]
−8
−6
−4
−2
0
2
4
Observed Outcome

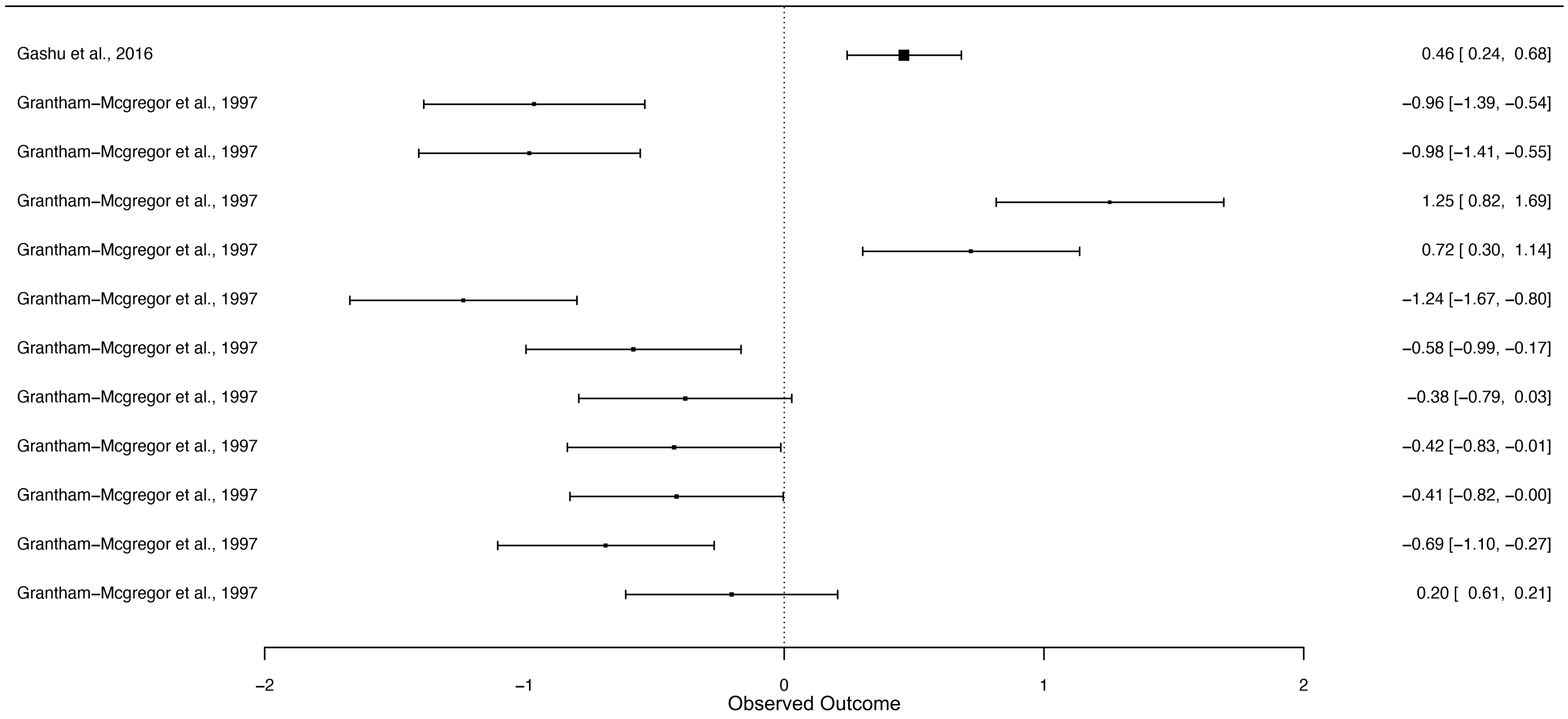

Gashu et al., 2016
0.46 [0.24, 0.68]
Grantham−Mcgregor et al., 1997
−0.96 [−1.39, −0.54]
Grantham−Mcgregor et al., 1997
−0.98 [−1.41, −0.55]
Grantham−Mcgregor et al., 1997
1.25 [0.82, 1.69]
Grantham−Mcgregor et al., 1997
0.72 [0.30, 1.14]
Grantham−Mcgregor et al., 1997
−1.24 [−1.67, −0.80]
Grantham−Mcgregor et al., 1997
−0.58 [−0.99, −0.17]
Grantham−Mcgregor et al., 1997
−0.38 [−0.79, 0.03]
Grantham−Mcgregor et al., 1997
−0.42 [−0.83, −0.01]
Grantham−Mcgregor et al., 1997
−0.41 [−0.82, −0.00]
Grantham−Mcgregor et al., 1997
−0.69 [−1.10, −0.27]
Grantham−Mcgregor et al., 1997
0.20 [0.61, 0.21]
−2
−1
0
1
2
Observed Outcome

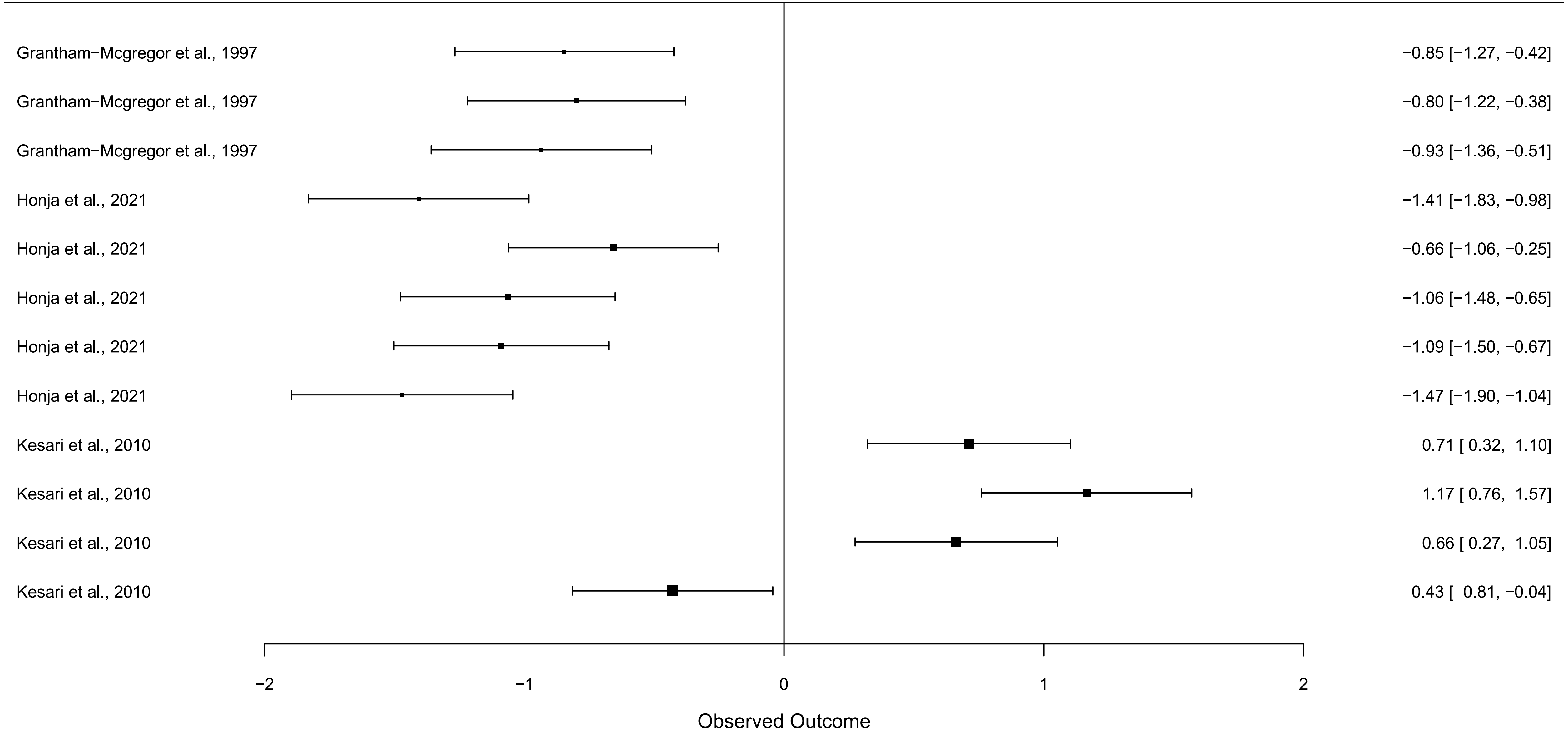

Grantham−Mcgregor et al., 1997
Grantham−Mcgregor et al., 1997
Grantham−Mcgregor et al., 1997
Honja et al., 2021
Honja et al., 2021
Honja et al., 2021
Honja et al., 2021
Honja et al., 2021
Kesari et al., 2010
Kesari et al., 2010
Kesari et al., 2010
Kesari et al., 2010
−0.85 [−1.27, −0.42]
−0.80 [−1.22, −0.38]
−0.93 [−1.36, −0.51]
−1.41 [−1.83, −0.98]
−0.66 [−1.06, −0.25]
−1.06 [−1.48, −0.65]
−1.09 [−1.50, −0.67]
−1.47 [−1.90, −1.04]
0.71 [0.32, 1.10]
1.17 [0.76, 1.57]
0.66 [0.27, 1.05]
0.43 [0.81, −0.04]
−2
−1
0
1
2
Observed Outcome

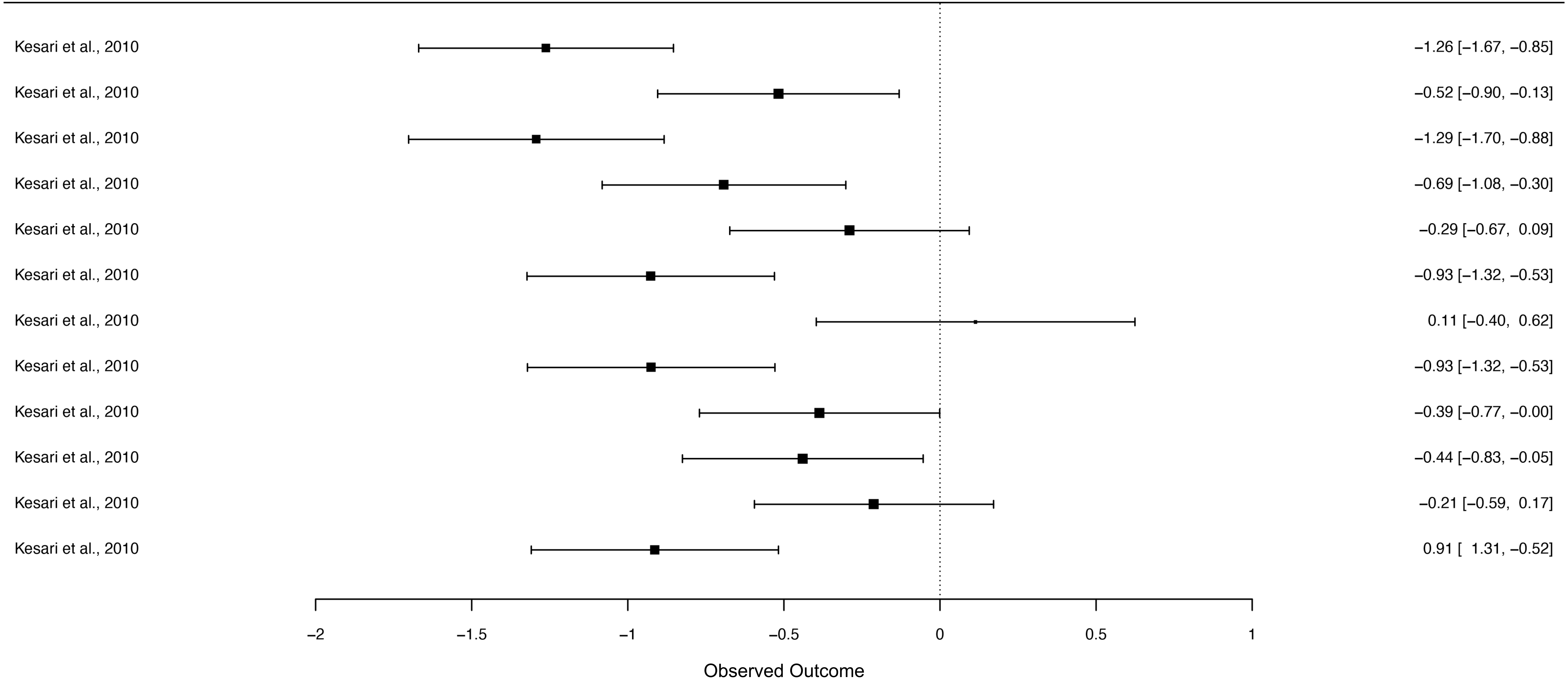

Kesari et al., 2010
−1.26 [−1.67, −0.85]
Kesari et al., 2010
−0.52 [−0.90, −0.13]
Kesari et al., 2010
−1.29 [−1.70, −0.88]
Kesari et al., 2010
−0.69 [−1.08, −0.30]
Kesari et al., 2010
−0.29 [−0.67, 0.09]
Kesari et al., 2010
−0.93 [−1.32, −0.53]
Kesari et al., 2010
0.11 [−0.40, 0.62]
Kesari et al., 2010
−0.93 [−1.32, −0.53]
Kesari et al., 2010
−0.39 [−0.77, −0.00]
Kesari et al., 2010
−0.44 [−0.83, −0.05]
Kesari et al., 2010
−0.21 [−0.59, 0.17]
Kesari et al., 2010
0.91 [1.31, −0.52]
−2
−1.5
−1
−0.5
0
0.5
1
Observed Outcome

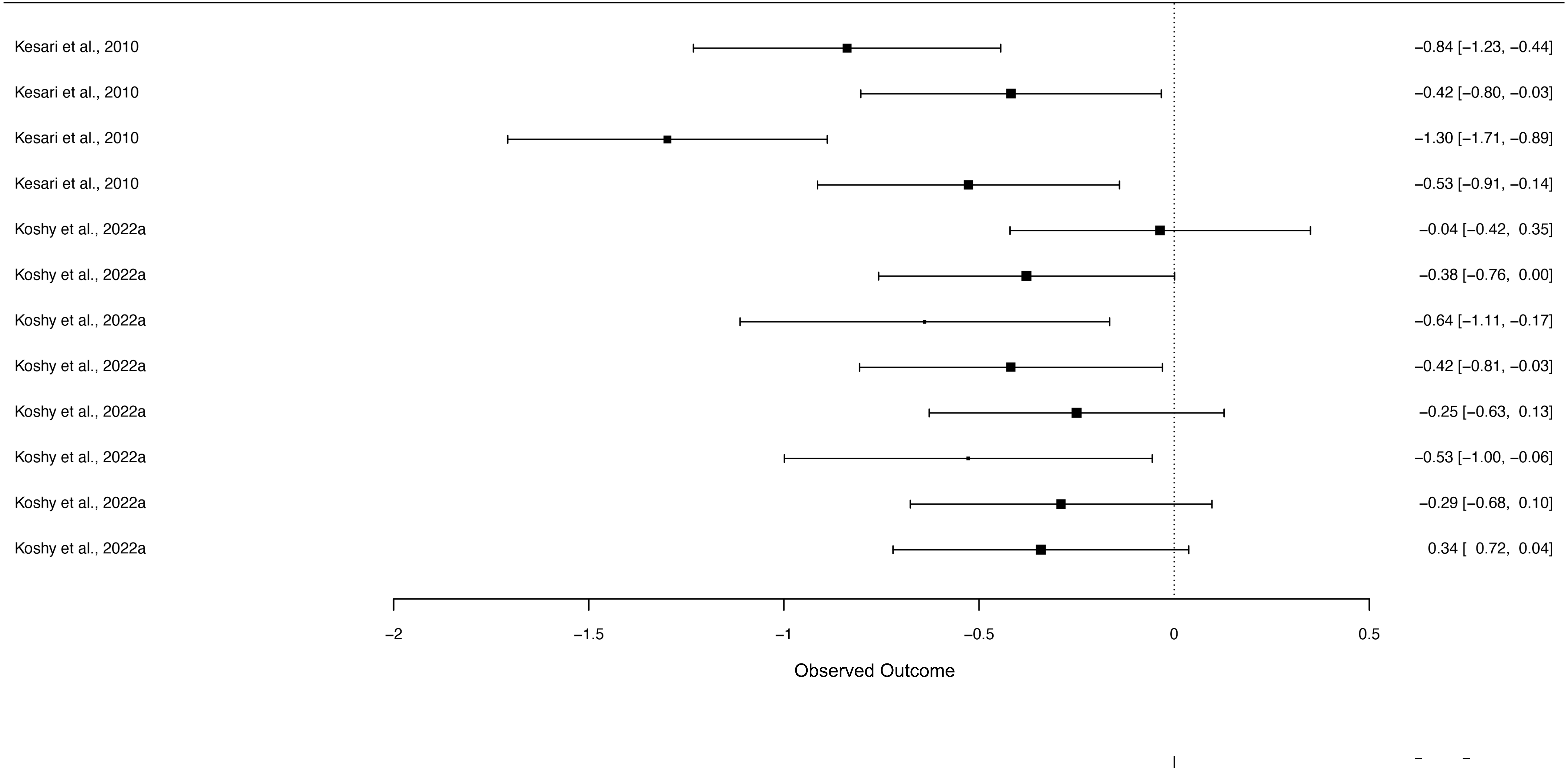
Kesari et al., 2010
−0.84 [−1.23, −0.44]
Kesari et al., 2010
−0.42 [−0.80, −0.03]
Kesari et al., 2010
−1.30 [−1.71, −0.89]
Kesari et al., 2010
−0.53 [−0.91, −0.14]
Koshy et al., 2022a
−0.04 [−0.42, 0.35]
Koshy et al., 2022a
−0.38 [−0.76, 0.00]
Koshy et al., 2022a
−0.64 [−1.11, −0.17]
Koshy et al., 2022a
−0.42 [−0.81, −0.03]
Koshy et al., 2022a
−0.25 [−0.63, 0.13]
Koshy et al., 2022a
−0.53 [−1.00, −0.06]
Koshy et al., 2022a
−0.29 [−0.68, 0.10]
Koshy et al., 2022a
0.34 [0.72, 0.04]
−2
−1.5
−1
−0.5
0
0.5
Observed Outcome

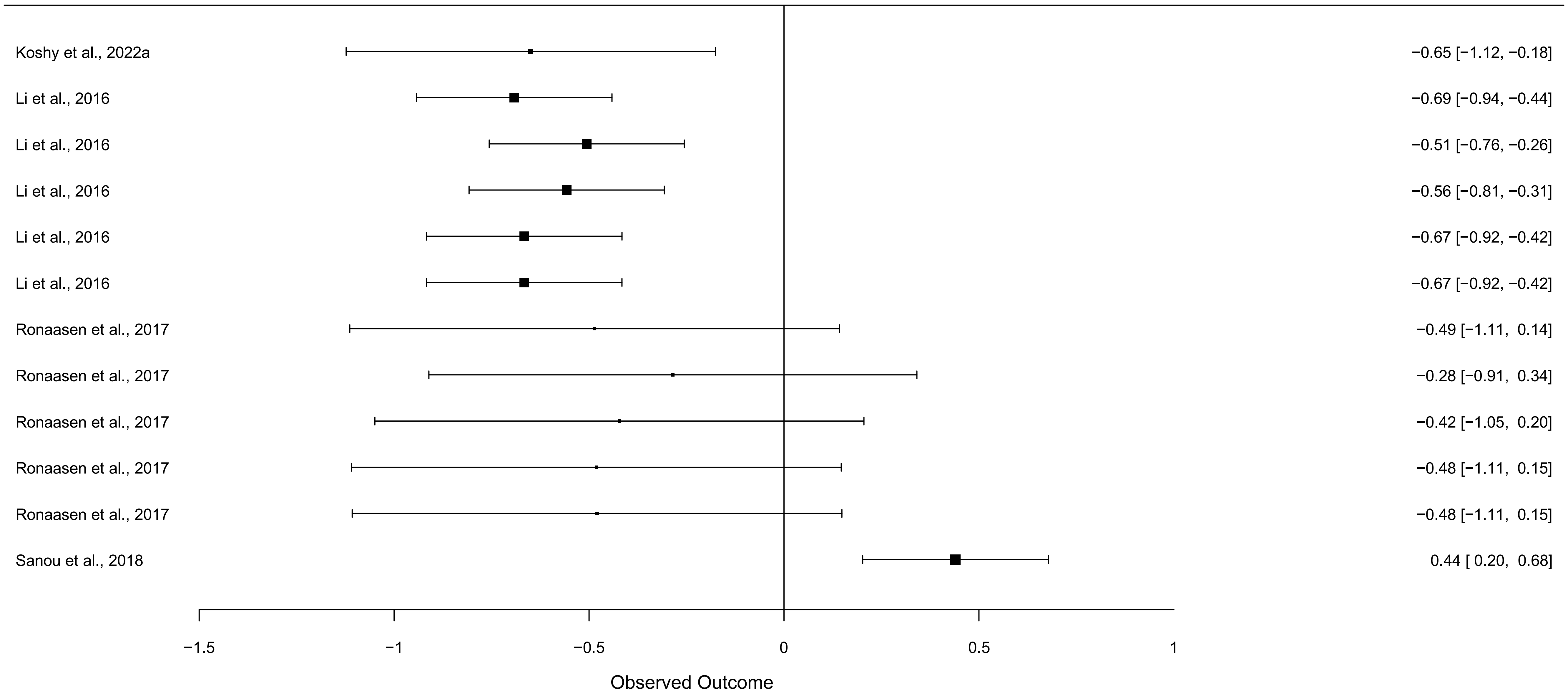
Koshy et al., 2022a
Li et al., 2016
Li et al., 2016
Li et al., 2016
Li et al., 2016
Li et al., 2016
Ronaasen et al., 2017
Ronaasen et al., 2017
Ronaasen et al., 2017
Ronaasen et al., 2017
Ronaasen et al., 2017
Sanou et al., 2018
−0.65 [−1.12, −0.18]
−0.69 [−0.94, −0.44]
−0.51 [−0.76, −0.26]
−0.56 [−0.81, −0.31]
−0.67 [−0.92, −0.42]
−0.67 [−0.92, −0.42]
−0.49 [−1.11, 0.14]
−0.28 [−0.91, 0.34]
−0.42 [−1.05, 0.20]
−0.48 [−1.11, 0.15]
−0.48 [−1.11, 0.15]
0.44 [0.20, 0.68]
−1.5
−1
−0.5
0
0.5
1
Observed Outcome

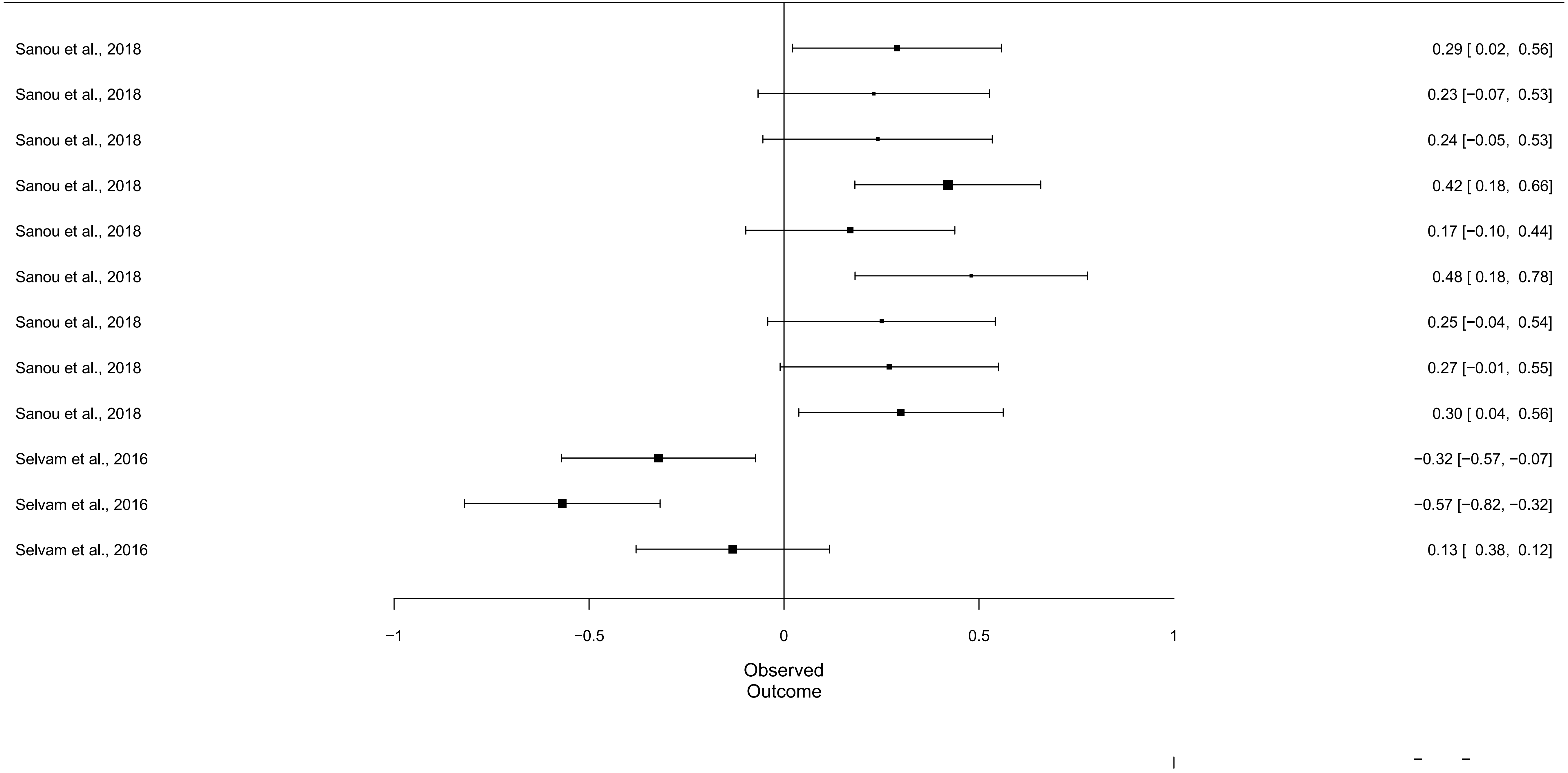
Sanou et al., 2018
0.29 [0.02, 0.56]
Sanou et al., 2018
0.23 [−0.07, 0.53]
Sanou et al., 2018
0.24 [−0.05, 0.53]
Sanou et al., 2018
0.42 [0.18, 0.66]
Sanou et al., 2018
0.17 [−0.10, 0.44]
Sanou et al., 2018
0.48 [0.18, 0.78]
Sanou et al., 2018
0.25 [−0.04, 0.54]
Sanou et al., 2018
0.27 [−0.01, 0.55]
Sanou et al., 2018
0.30 [0.04, 0.56]
Selvam et al., 2016
−0.32 [−0.57, −0.07]
Selvam et al., 2016
−0.57 [−0.82, −0.32]
Selvam et al., 2016
0.13 [0.38, 0.12]
−1
−0.5
0
0.5
1
Observed
Outcome

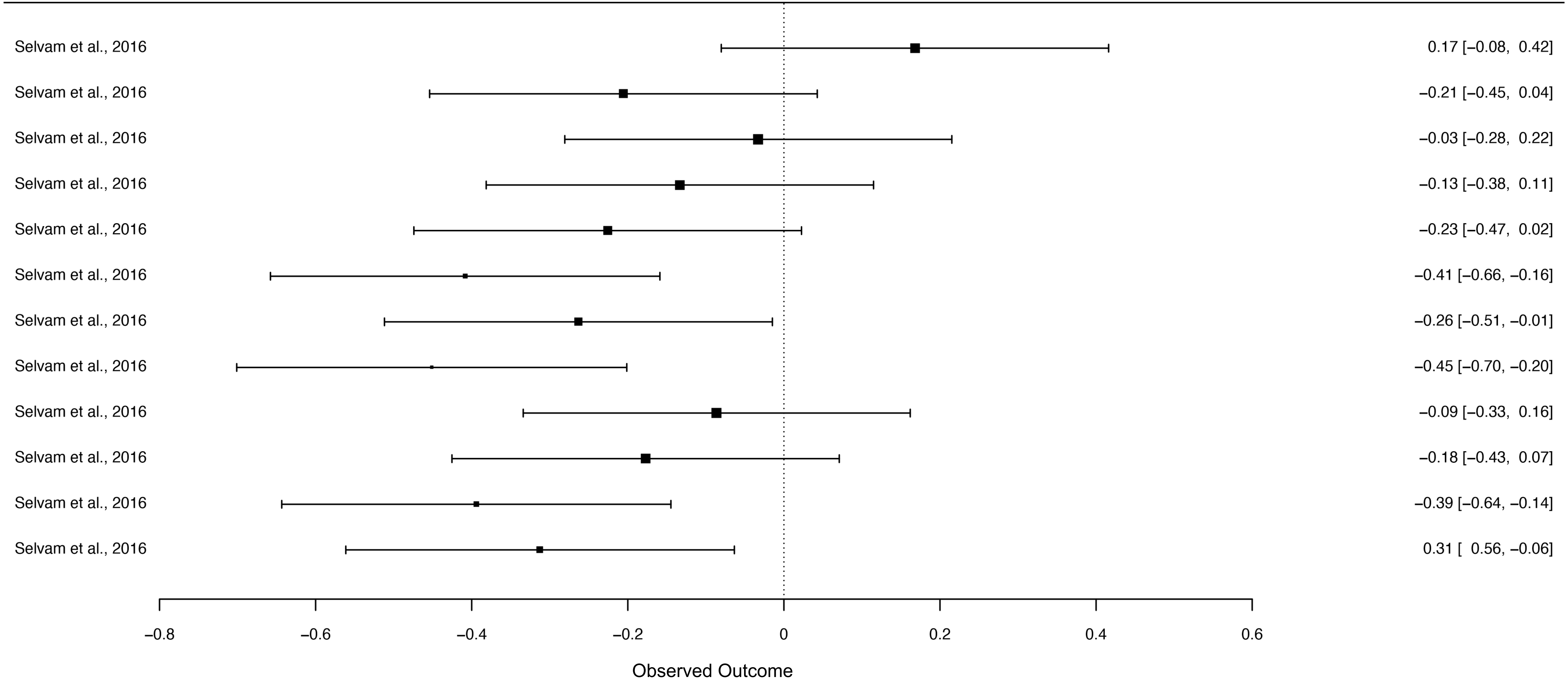

Selvam et al., 2016
0.17 [–0.08, 0.42]
Selvam et al., 2016
–0.21 [–0.45, 0.04]
Selvam et al., 2016
–0.03 [–0.28, 0.22]
Selvam et al., 2016
–0.13 [–0.38, 0.11]
Selvam et al., 2016
–0.23 [–0.47, 0.02]
Selvam et al., 2016
–0.41 [–0.66, –0.16]
Selvam et al., 2016
–0.26 [–0.51, –0.01]
Selvam et al., 2016
–0.45 [–0.70, –0.20]
Selvam et al., 2016
–0.09 [–0.33, 0.16]
Selvam et al., 2016
–0.18 [–0.43, 0.07]
Selvam et al., 2016
–0.39 [–0.64, –0.14]
Selvam et al., 2016
0.31 [0.56, –0.06]
–0.8
–0.6
–0.4
–0.2
0
0.2
0.4
0.6
Observed Outcome

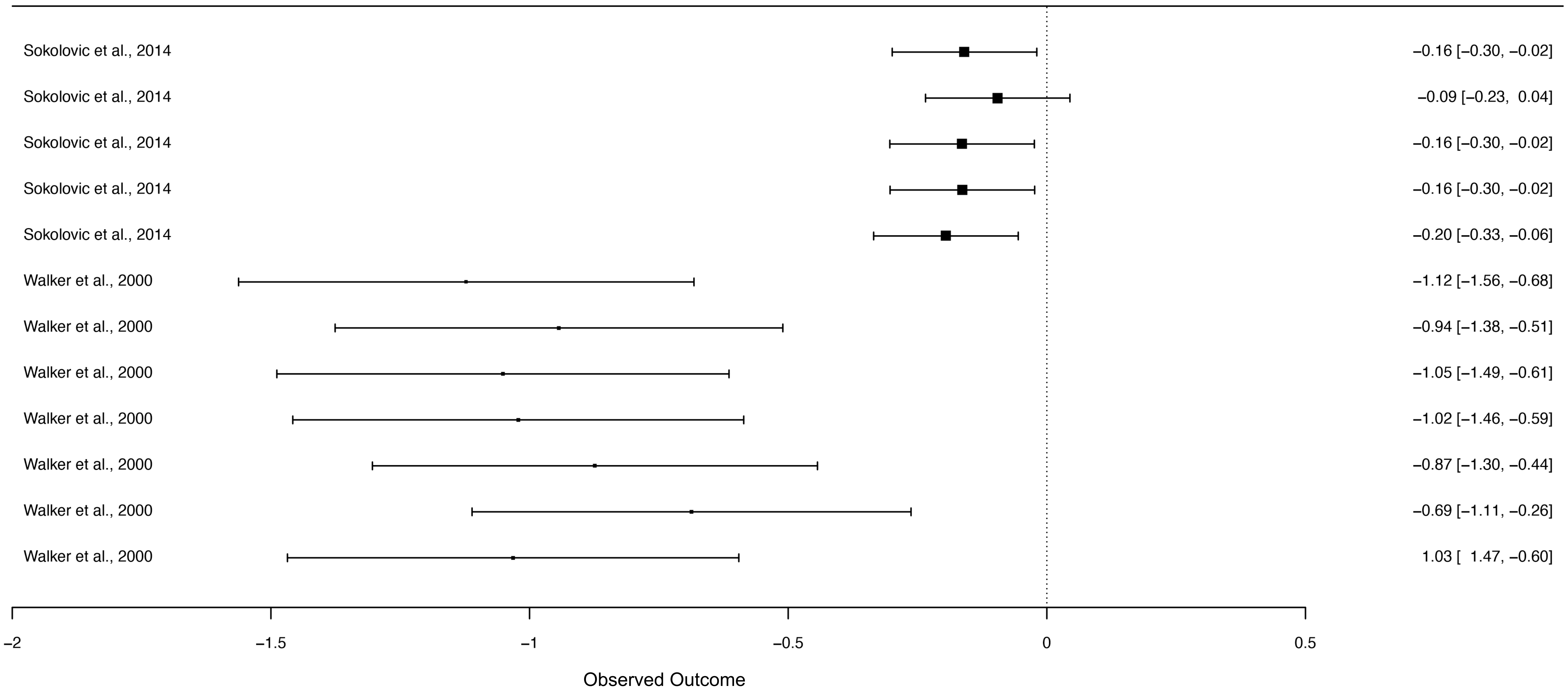
Sokolovic et al., 2014
−0.16 [−0.30, −0.02]
Sokolovic et al., 2014
−0.09 [−0.23, 0.04]
Sokolovic et al., 2014
−0.16 [−0.30, −0.02]
Sokolovic et al., 2014
−0.16 [−0.30, −0.02]
Sokolovic et al., 2014
−0.20 [−0.33, −0.06]
Walker et al., 2000
−1.12 [−1.56, −0.68]
Walker et al., 2000
−0.94 [−1.38, −0.51]
Walker et al., 2000
−1.05 [−1.49, −0.61]
Walker et al., 2000
−1.02 [−1.46, −0.59]
Walker et al., 2000
−0.87 [−1.30, −0.44]
Walker et al., 2000
−0.69 [−1.11, −0.26]
Walker et al., 2000
1.03 [1.47, −0.60]
−2
−1.5
−1
−0.5
0
0.5
Observed Outcome

– –

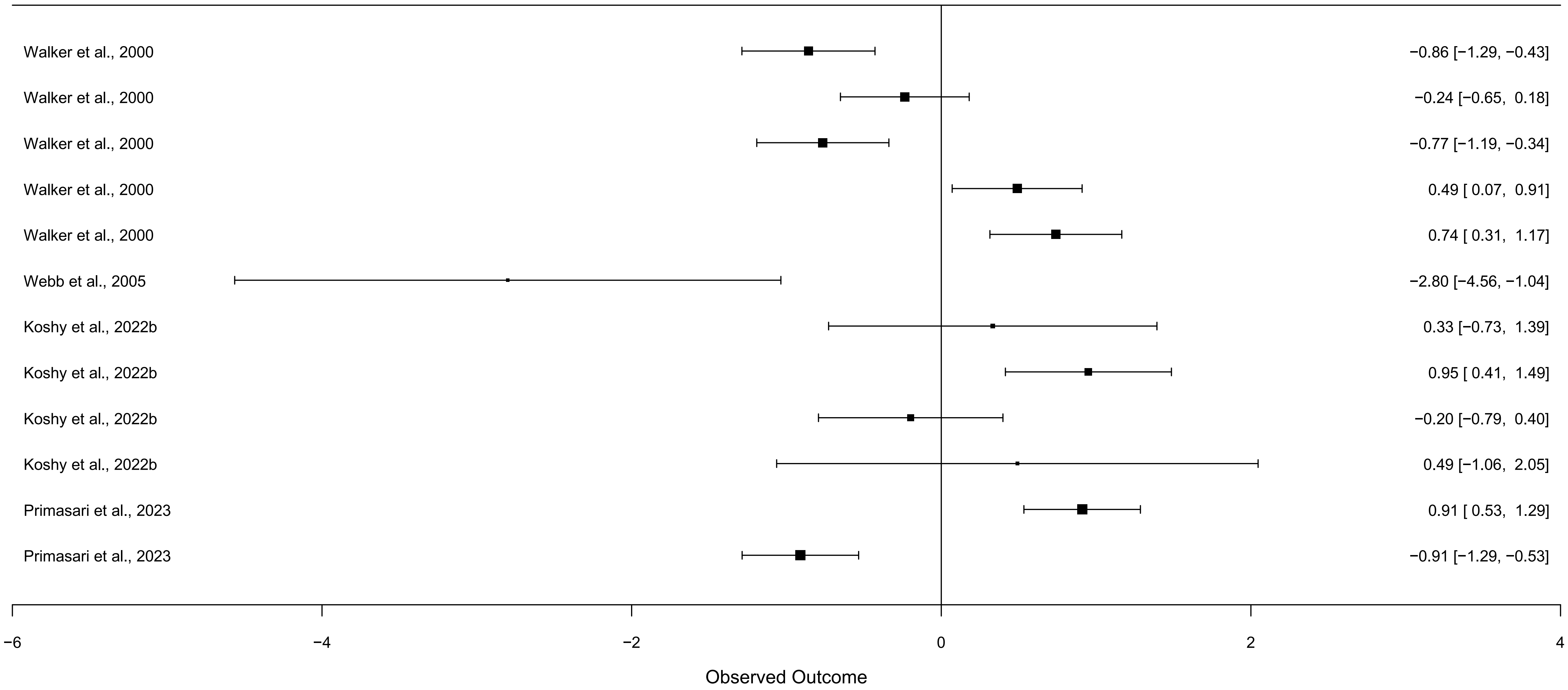
Walker et al., 2000
−0.86 [−1.29, −0.43]
Walker et al., 2000
−0.24 [−0.65, 0.18]
Walker et al., 2000
−0.77 [−1.19, −0.34]
Walker et al., 2000
0.49 [0.07, 0.91]
Walker et al., 2000
0.74 [0.31, 1.17]
Webb et al., 2005
−2.80 [−4.56, −1.04]
Koshy et al., 2022b
0.33 [−0.73, 1.39]
Koshy et al., 2022b
0.95 [0.41, 1.49]
Koshy et al., 2022b
−0.20 [−0.79, 0.40]
Koshy et al., 2022b
0.49 [−1.06, 2.05]
Primasari et al., 2023
0.91 [0.53, 1.29]
Primasari et al., 2023
−0.91 [−1.29, −0.53]
−6
−4
−2
0
2
4
Observed Outcome

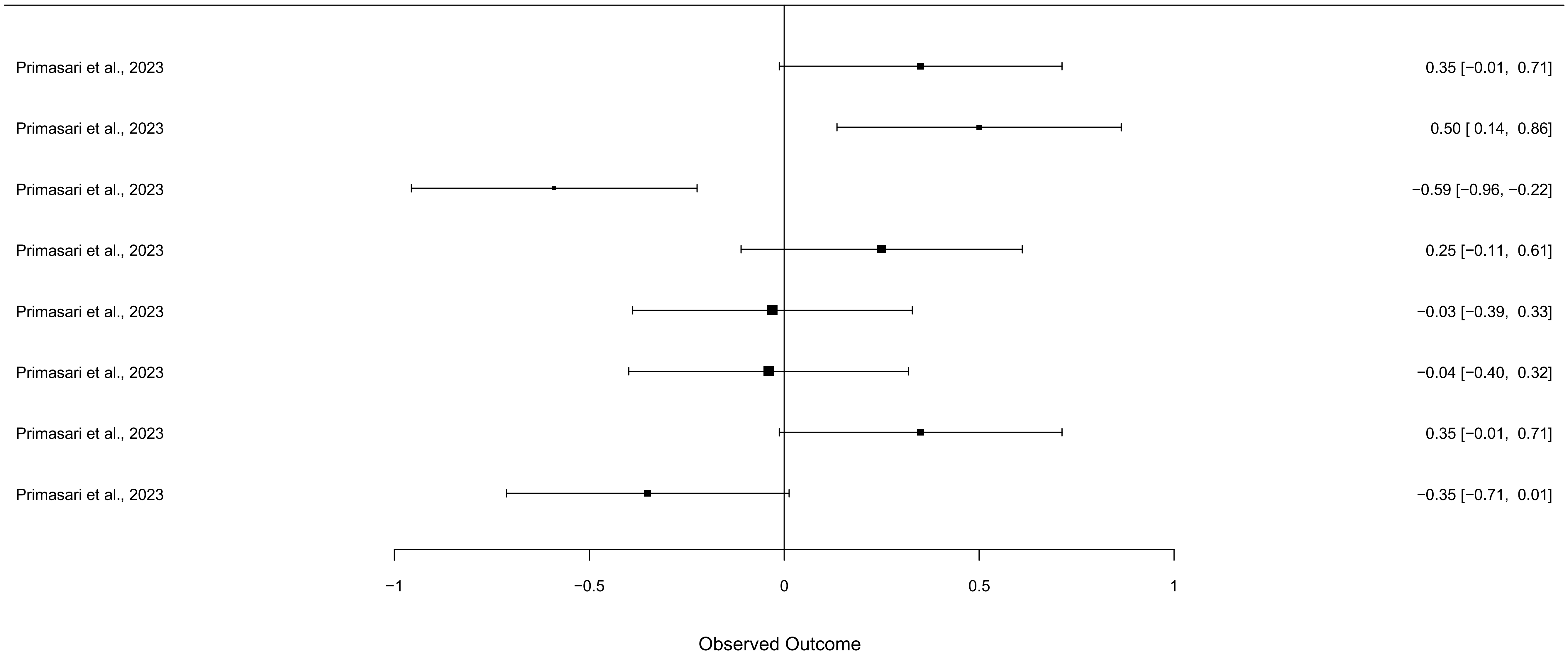
Primasari et al., 2023
0.35 [−0.01, 0.71]
Primasari et al., 2023
0.50 [0.14, 0.86]
Primasari et al., 2023
−0.59 [−0.96, −0.22]
Primasari et al., 2023
0.25 [−0.11, 0.61]
Primasari et al., 2023
−0.03 [−0.39, 0.33]
Primasari et al., 2023
−0.04 [−0.40, 0.32]
Primasari et al., 2023
0.35 [−0.01, 0.71]
Primasari et al., 2023
−0.35 [−0.71, 0.01]
−1
−0.5
0
0.5
1
Observed Outcome

Figure S2. Funnel plot of the main model (including all studies).

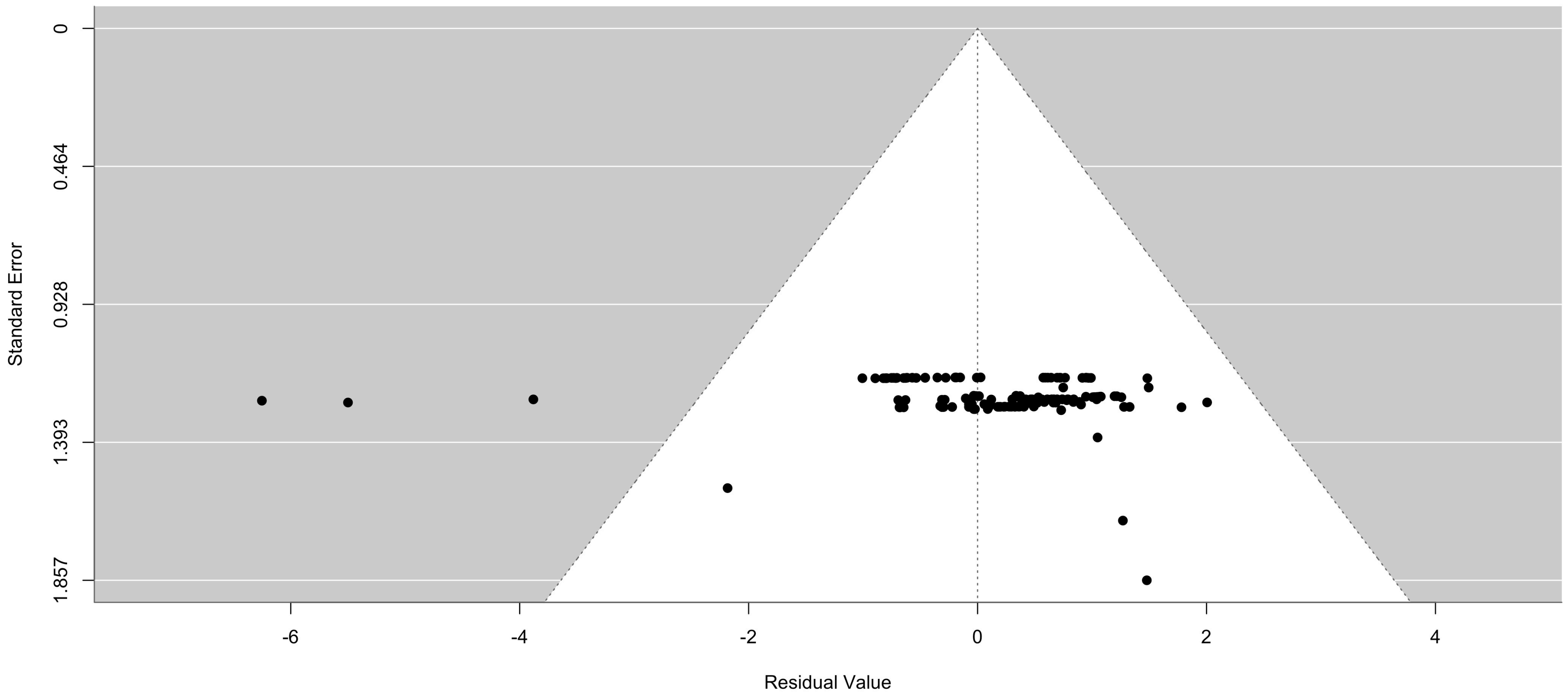

Sub-analyses of direct effect on cognitive development in childhood stunting (3.1.1)

Table S5. Coefficients of all sub-domain models

Domain	Coefficient	Estimate	SE	Z value	p value	95% CI Lower	95% CI Upper	Significance
Academic Skills	intercept	0.4621	0.8083	0.5717	0.5675	-1.1221	2.0463	
Academic Skills	Age	-1.2948	0.9675	-1.3384	0.1808	-3.1911	0.6014	
Academic Skills	Region Asia	1.0867	0.8786	1.2369	0.2161	-0.6353	2.8088	
Academic Skills	Region North America	-0.0265	0.9035	-0.0293	0.9766	-1.7973	1.7444	
**Socioemotional Development**	**intercept**	**-1.2277**	**0.4115**	**-2.9838**	**0.0028**	**-2.0341**	**-0.4212**	**
**Socioemotional Development**	**Age**	**0.7680**	**0.3773**	**2.0353**	**0.0418**	**0.0284**	**1.5075**	*
**Socioemotional Development**	**Region Asia**	**0.9440**	**0.4139**	**2.2804**	**0.0226**	**0.1327**	**1.7553**	*
**Cognition**	intercept	-3.0755	1.3009	-2.3641	0.0181	-5.6252	-0.5257	*
Cognition	Age	2.3870	1.3906	1.7165	0.0861	-0.3385	5.1125	
Cognition	Region Asia	-0.1351	1.3439	-0.1006	0.9199	-2.7692	2.4989	
Cognition	Region North America	0.6233	1.9242	0.3239	0.7460	-3.1481	4.3947	
Motor Skills	intercept	0.6645	0.6328	1.0501	0.2937	-0.5758	1.9047	
Motor Skills	Age	-0.0800	0.5856	-0.1366	0.8914	-1.2278	1.0678	
Motor Skills	Region Asia	-0.7959	0.5851	-1.3603	0.1737	-1.9426	0.3509	
Memory	intercept	-0.2993	0.4055	-0.7381	0.4605	-1.0941	0.4955	
Memory	Region Asia	-0.1882	0.5756	-0.3269	0.7437	-1.3164	0.9400	
Memory	Region North America	-0.2167	0.5761	-0.3762	0.7068	-1.3458	0.9124	
**Executive Function**	**intercept**	**-0.6868**	**0.2885**	**-2.3806**	**0.0173**	**-1.2523**	**-0.1213**	*
**Executive Function**	**Region Asia**	**1.5293**	**0.3323**	**4.6027**	**0.0000**	**0.8781**	**2.1805**	***
Oral Language	intercept	-0.3907	0.4697	-0.8317	0.4056	-1.3114	0.5300	
Oral Language	Age	-0.0951	0.2351	-0.4046	0.6858	-0.5558	0.3656	
Oral Language	Region Asia	0.1358	0.4385	0.3096	0.7568	-0.7236	0.9951	
Oral Language	Region North America	-0.4661	0.4887	-0.9538	0.3402	-1.4240	0.4917	
**Intelligence**	**intercept**	**-0.8495**	**0.3015**	**-2.8173**	**0.0048**	**-1.4405**	**-0.2585**	**

Intelligence	Age	-0.1843	0.1413	-1.3044	0.1921	-0.4613	0.0926	
**Intelligence**	**Region Asia**	**0.6185**	**0.3059**	**2.0218**	**0.0432**	**0.0189**	**1.2181**	*
**Visuospatial**	**intercept**	**0.4901**	**0.2141**	**2.2891**	**0.0221**	**0.0705**	**0.9097**	*
**Visuospatial**	**Region Asia**	**-0.9296**	**0.2907**	**-3.1974**	**0.0014**	**-1.4994**	**-0.3598**	**

Table S6. Heterogeneity of all sub-domain models

**Domain**	**QE**	**df**	**p value**	**Significance**	**tau2 (sigma21) Study ID**	**tau2 (sigma22) ES**	**I2_total**
**Academic Skills**	**788.2301**	**16**	**0**	***	**0.4568**	**0.1839**	**96.1980**
**Socioemotional Development**	**106.6794**	**18**	**0**	***	**0.1164**	**0.0228**	**62.9427**
**Cognition**	**1829.7509**	**28**	**0**	***	**4.6811**	**0.2315**	**96.6838**
**Motor Skills**	**117.6717**	**18**	**0**	***	**0.0617**	**0.1849**	**86.3500**
**Memory**	**168.8557**	**23**	**0**	***	**0.3127**	**0.0303**	**90.5542**
Executive Function	3.6880	2	0.1582		0.0023	0.0341	46.3089
**Oral Language**	**24.4115**	**7**	**0.0010**	***	**0.0352**	**0.0275**	**64.6394**
Intelligence	20.0262	12	0.0666		0.0586	0	58.4995
Visuospatial	0	0	1.0000		0	0	0

Table S7. Moderators of all sub-domain models

Domain	QM	df	p value	Significance
Academic Skills	3.0616	3	0.3822	
**Socioemotional Development**	**6.7965**	**2**	**0.0334**	*
Cognition	3.8023	3	0.2836	
Motor Skills	5.0522	2	0.0800	
Memory	0.1676	2	0.9196	
**Executive Function**	**21.1847**	**1**	**0.0000**	***
Oral Language	5.0150	3	0.1707	
**Intelligence**	**6.1278**	**2**	**0.0467**	*
**Visuospatial**	**10.2231**	**1**	**0.0014**	**

Table S8. Publication bias test of all sub-domain models

Domain	Z value	p value	Significance
Academic Skills	-2.0687	0.0533	
Socioemotional Development	1.1058	0.2826	
Cognition	0.3947	0.6959	
**Motor Skills**	**2.9463**	**0.0083**	**
**Memory**	**-5.3278**	**0**	***
Executive Function	-1.6253	0.2456	
Oral Language	-0.8996	0.3918	
Intelligence	-1.8160	0.0925	

Table S9. Variance Components

	β	sqrt	N levels	Fixed	factor
Test for Residual Heterogeneity					
	$Q_E(117) = 55738.8561$, $p<.001$				
sigma^2.1	0.5677	0.7535	23	No	Study ID
sigma^2.2	0.8846	0.9405	129	No	Effect Size

– –

Figure S3. Forest plot of the 125 effect sizes along with their 95% confidence intervals. *(Moderator effect on cognitive development in childhood stunting (3.2)).*

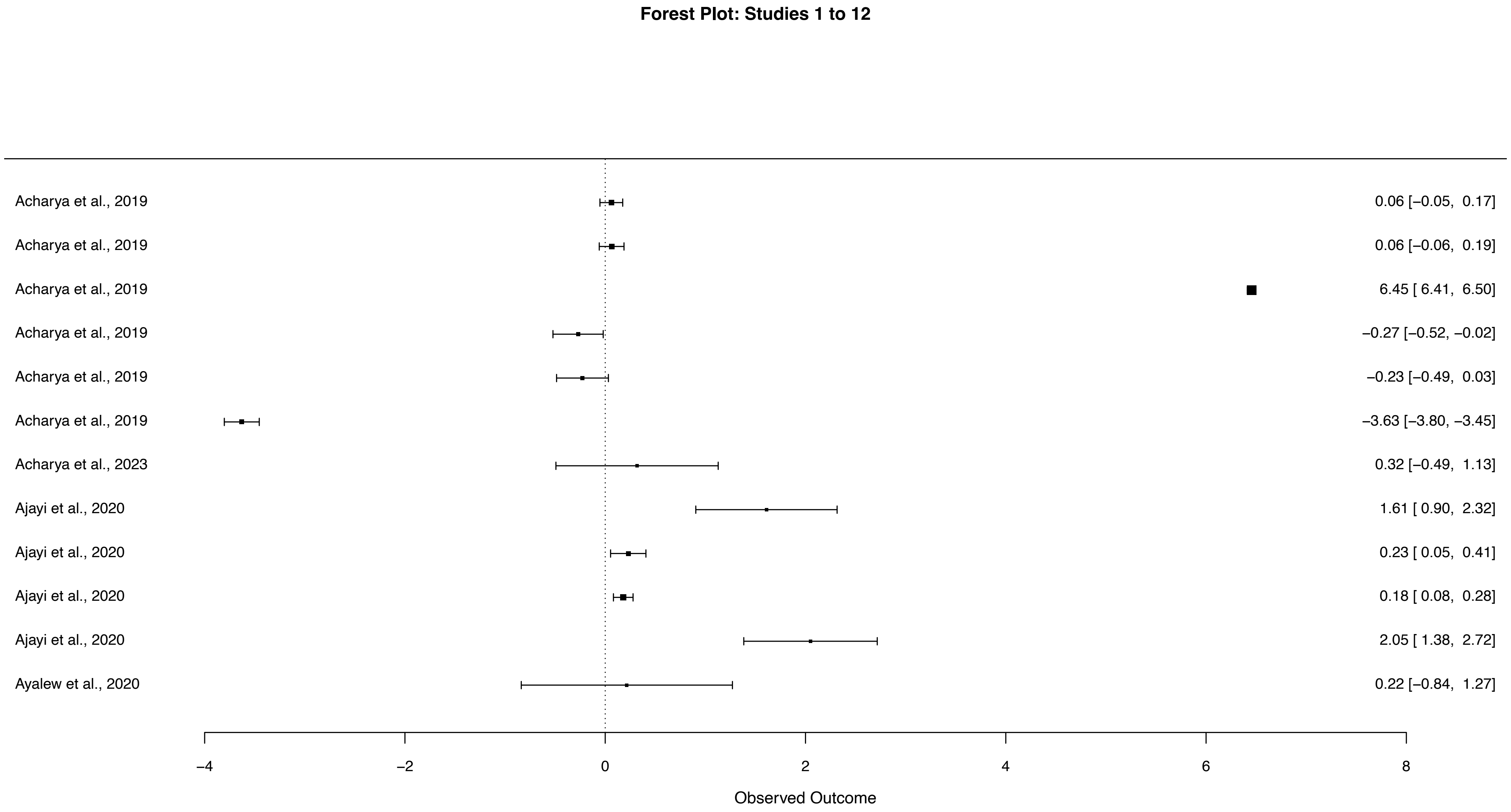

Forest Plot: Studies 13 to 24

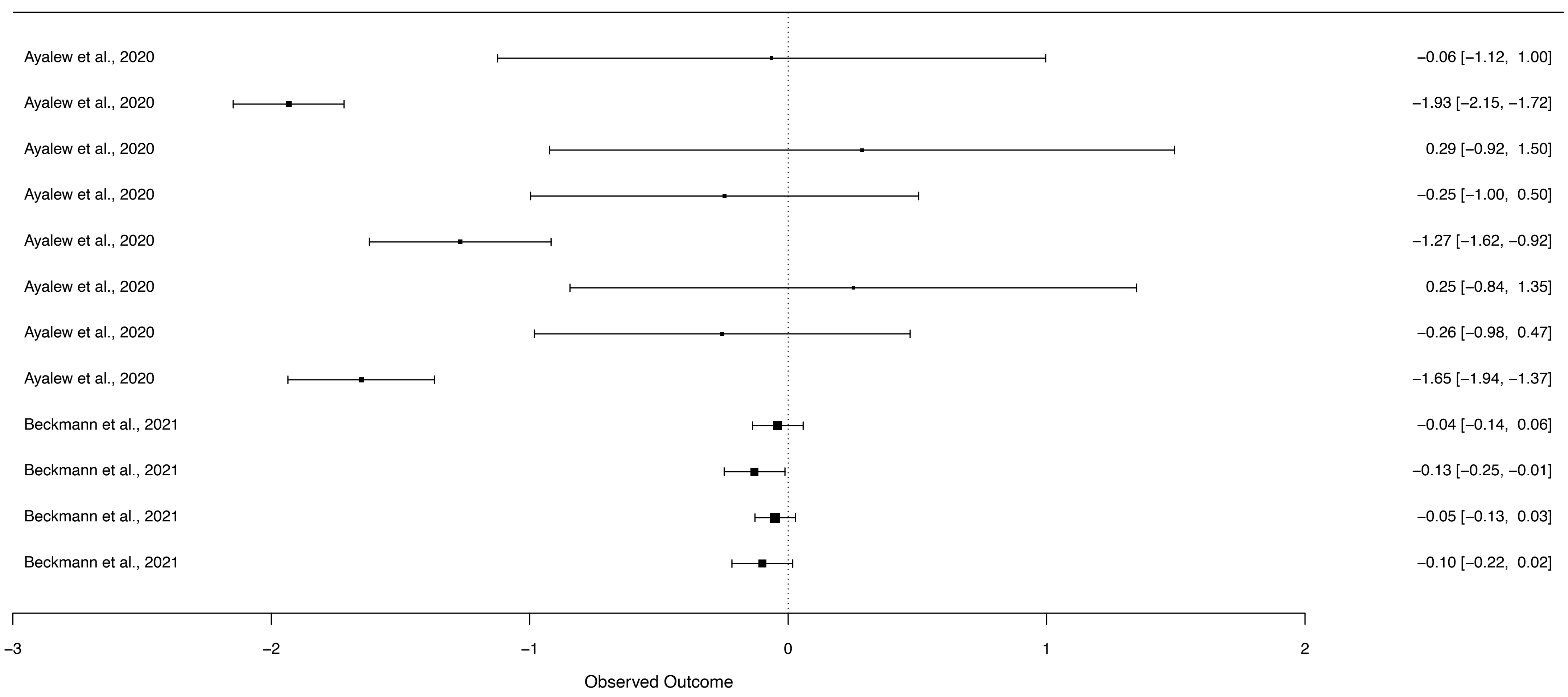

Forest Plot: Studies 25 to 36

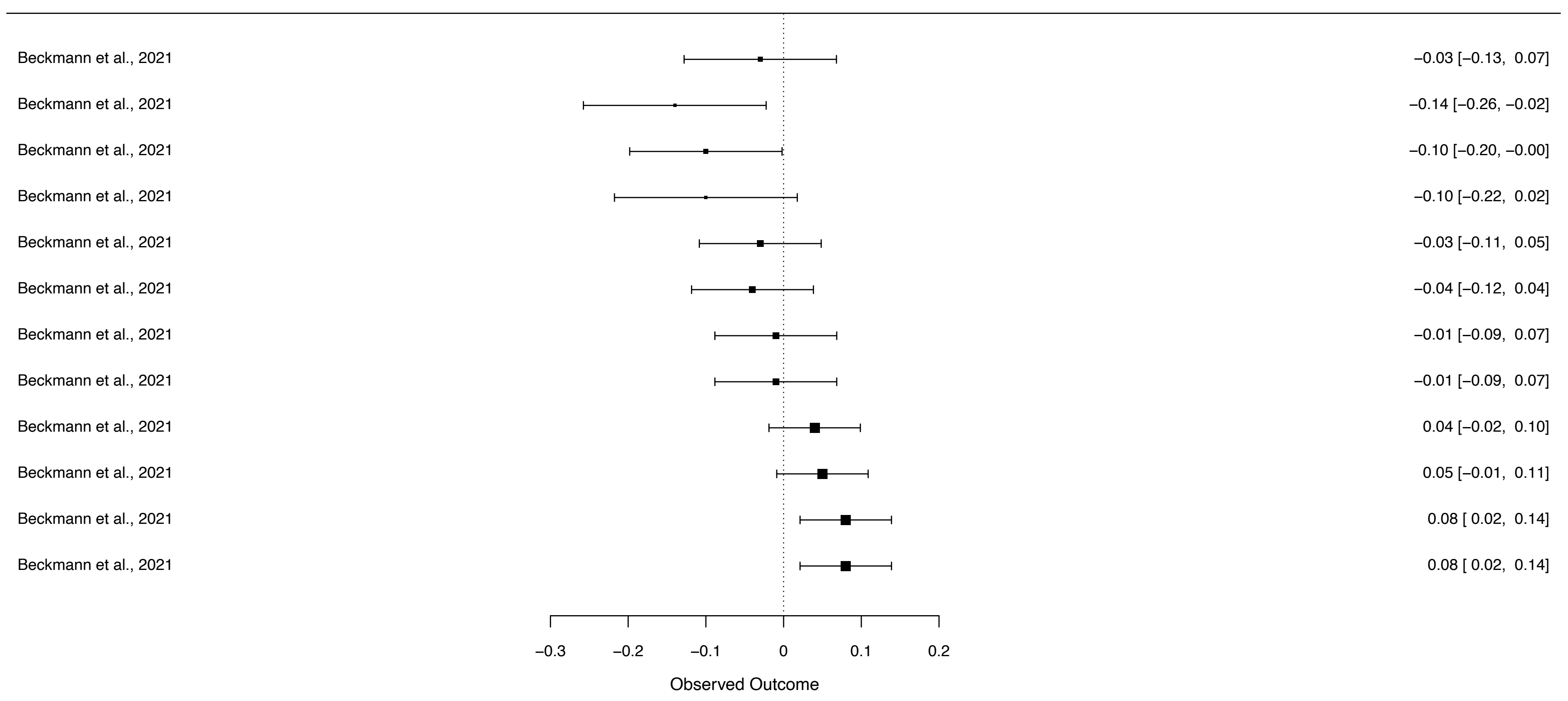

Forest Plot: Studies 37 to 48

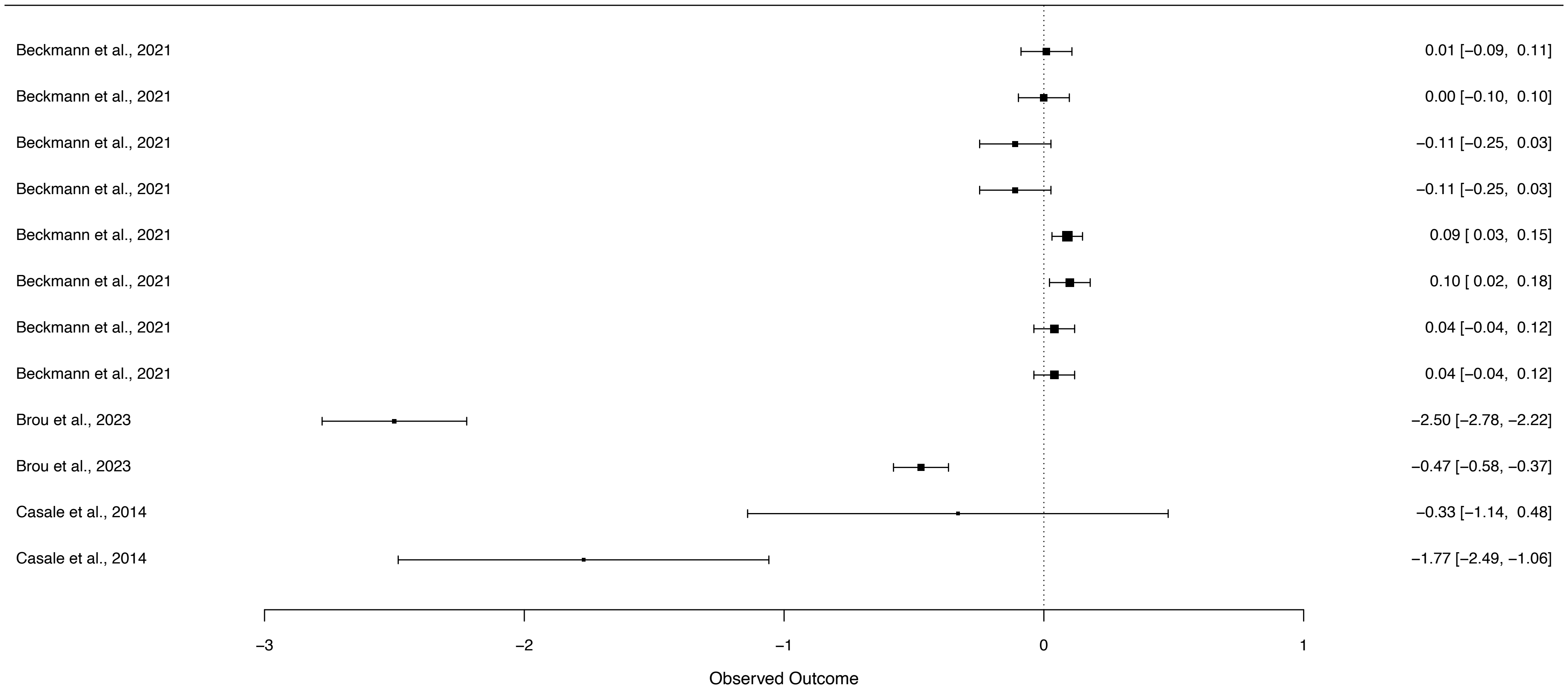

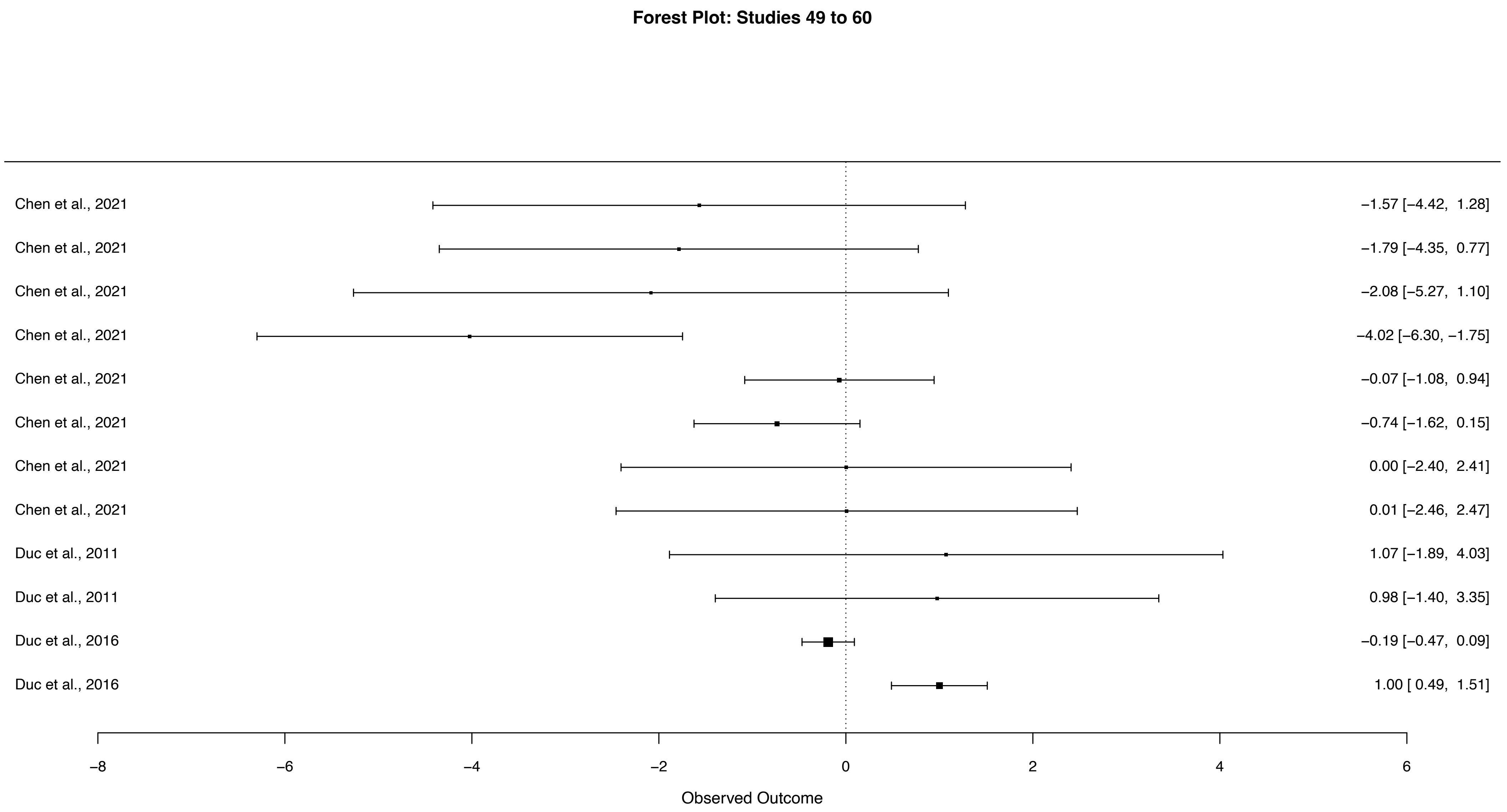
Forest Plot: Studies 49 to 60
Chen et al., 2021
−1.57 [−4.42, 1.28]
Chen et al., 2021
−1.79 [−4.35, 0.77]
Chen et al., 2021
−2.08 [−5.27, 1.10]
Chen et al., 2021
−4.02 [−6.30, −1.75]
Chen et al., 2021
−0.07 [−1.08, 0.94]
Chen et al., 2021
−0.74 [−1.62, 0.15]
Chen et al., 2021
0.00 [−2.40, 2.41]
Chen et al., 2021
0.01 [−2.46, 2.47]
Duc et al., 2011
1.07 [−1.89, 4.03]
Duc et al., 2011
0.98 [−1.40, 3.35]
Duc et al., 2016
−0.19 [−0.47, 0.09]
Duc et al., 2016
1.00 [0.49, 1.51]
−8
−6
−4
−2
0
2
4
6
Observed Outcome

Forest Plot: Studies 61 to 72

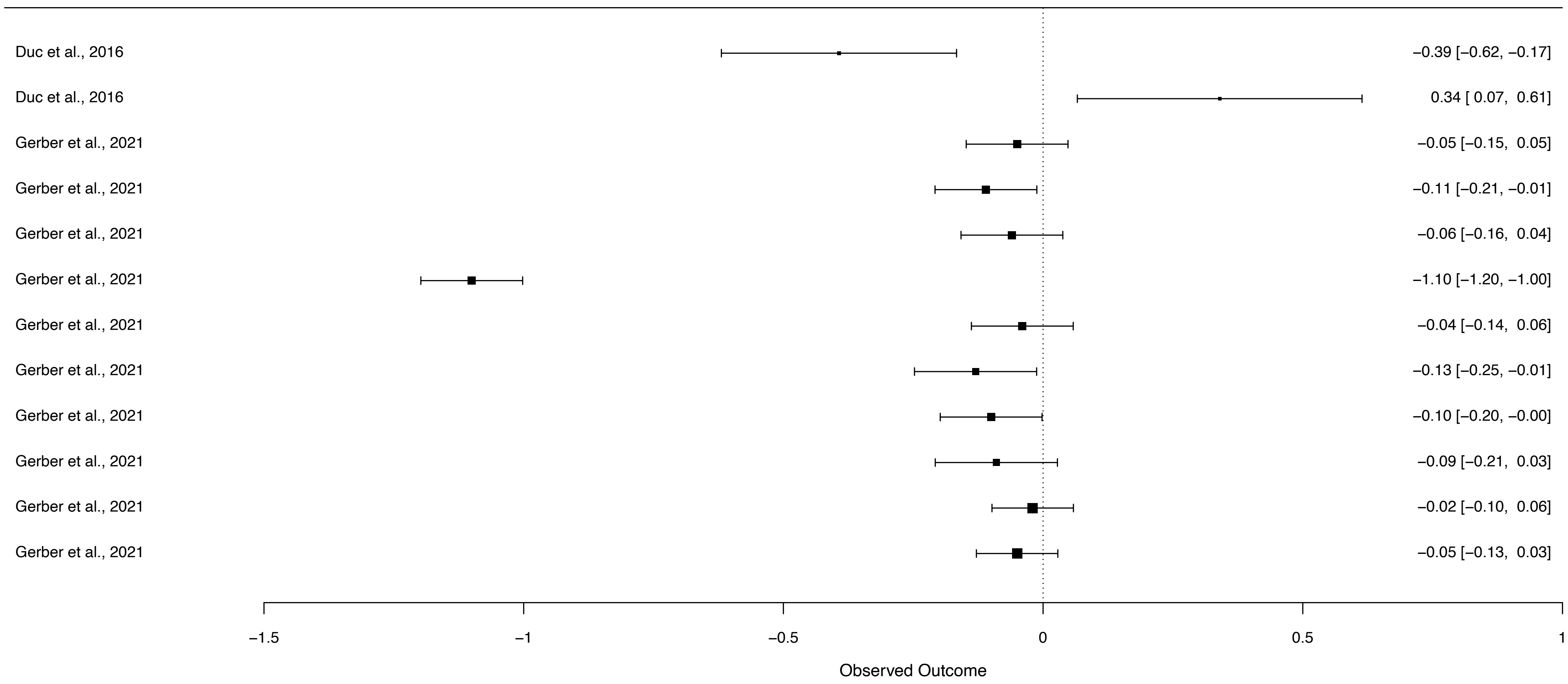

Forest Plot: Studies 73 to 84

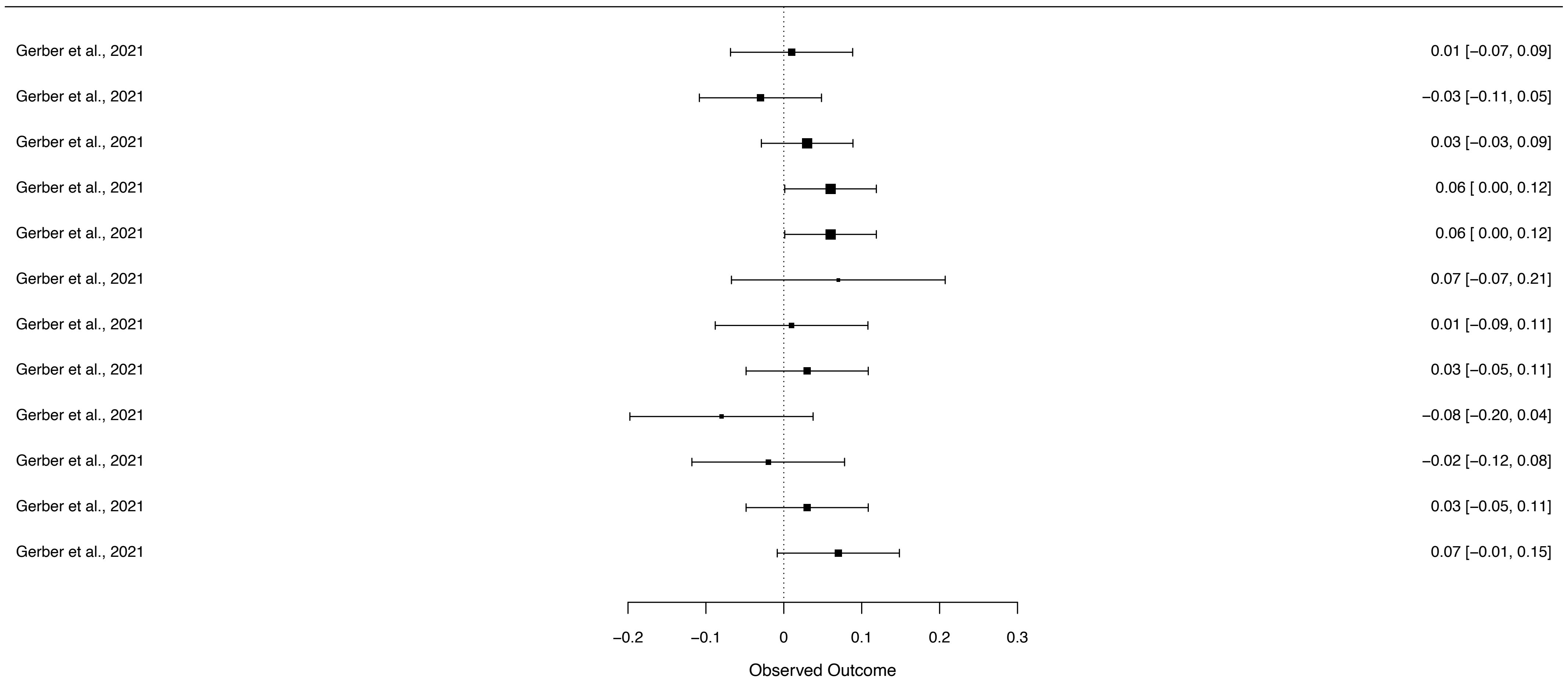

Forest Plot: Studies 85 to 96

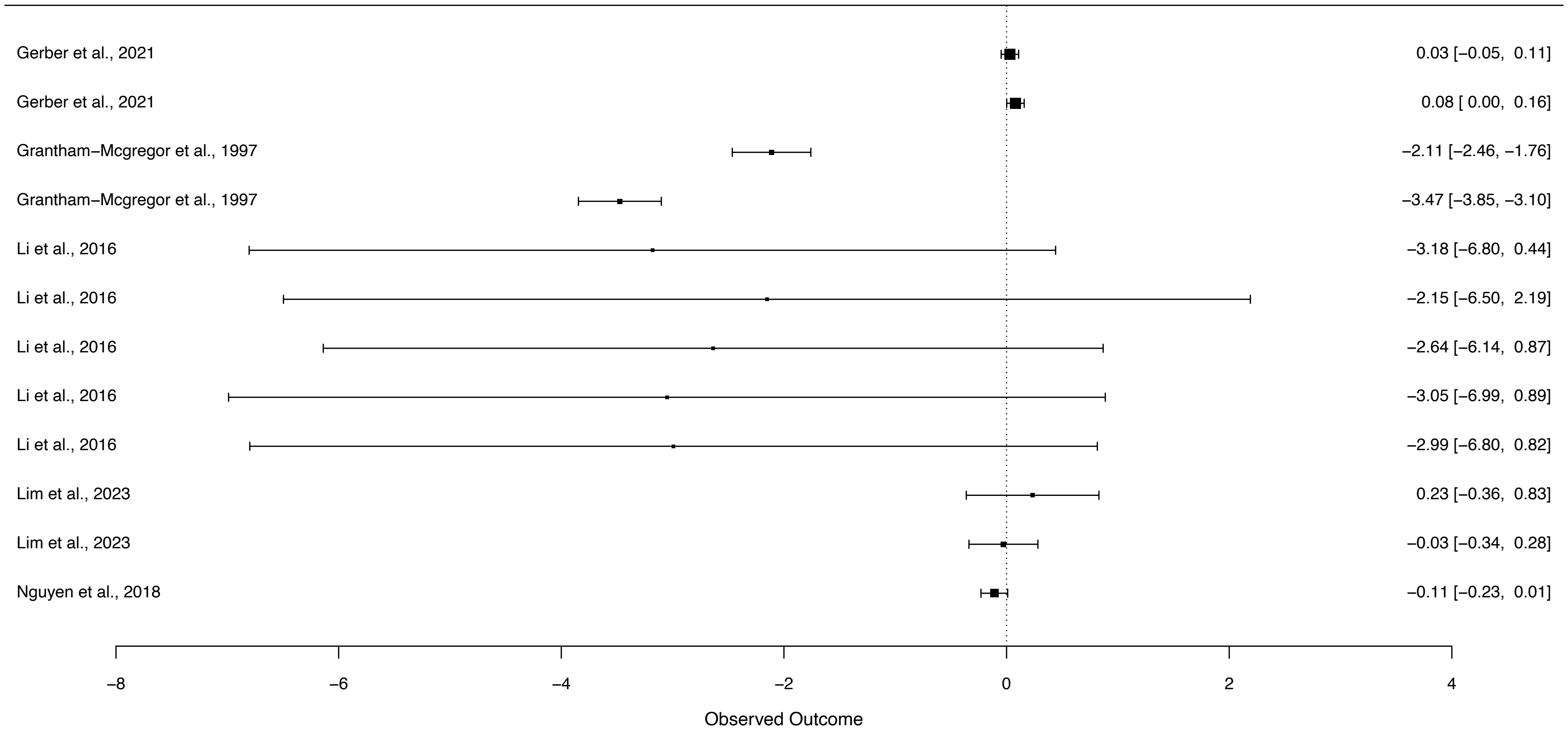

Forest Plot: Studies 97 to 108

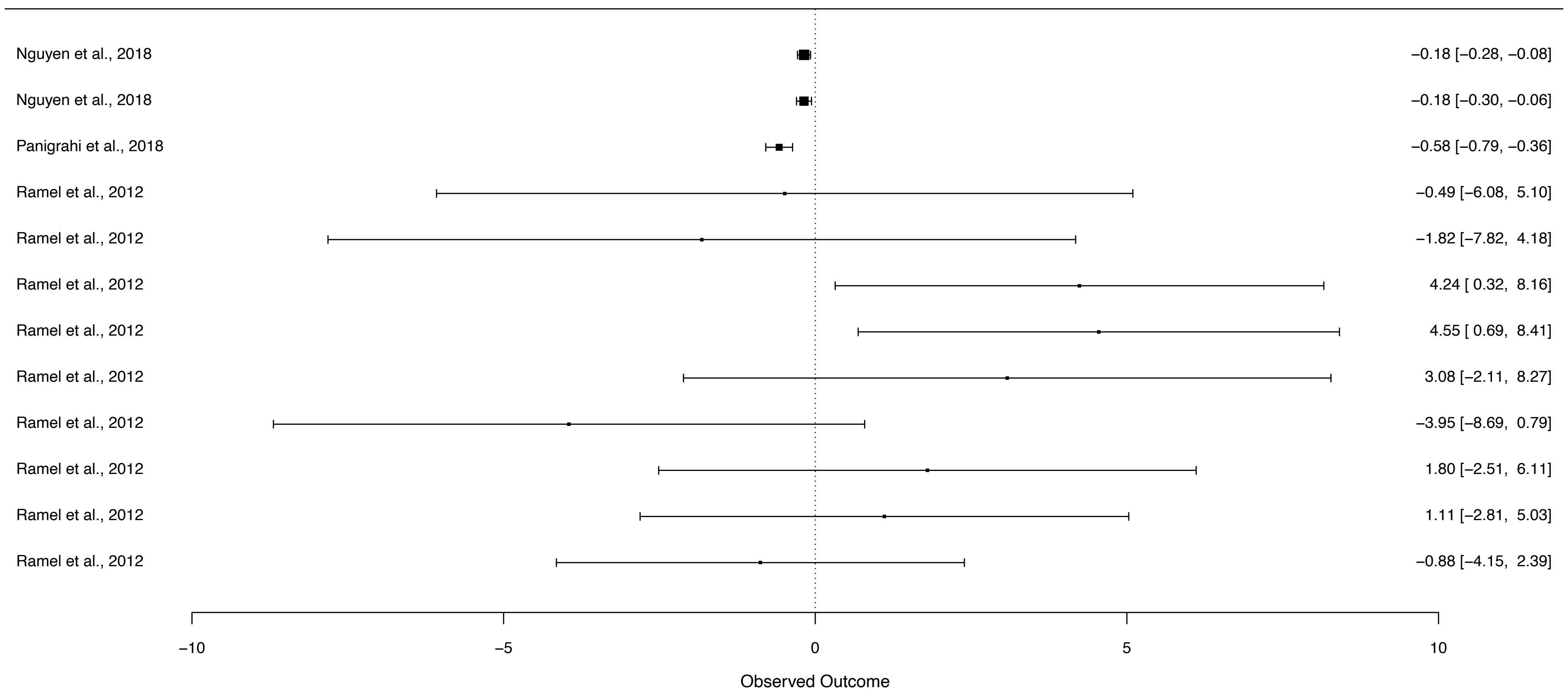

Forest Plot: Studies 109 to 120

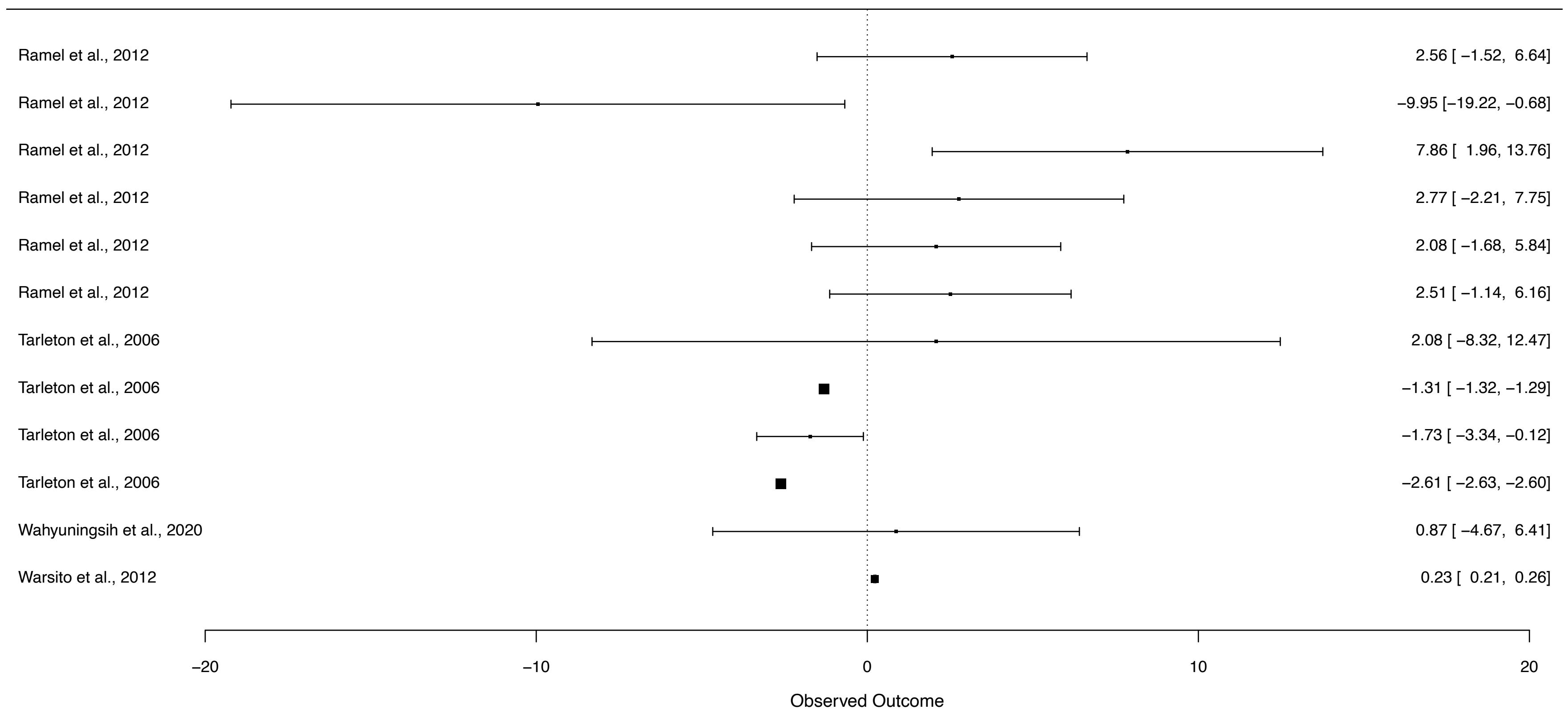

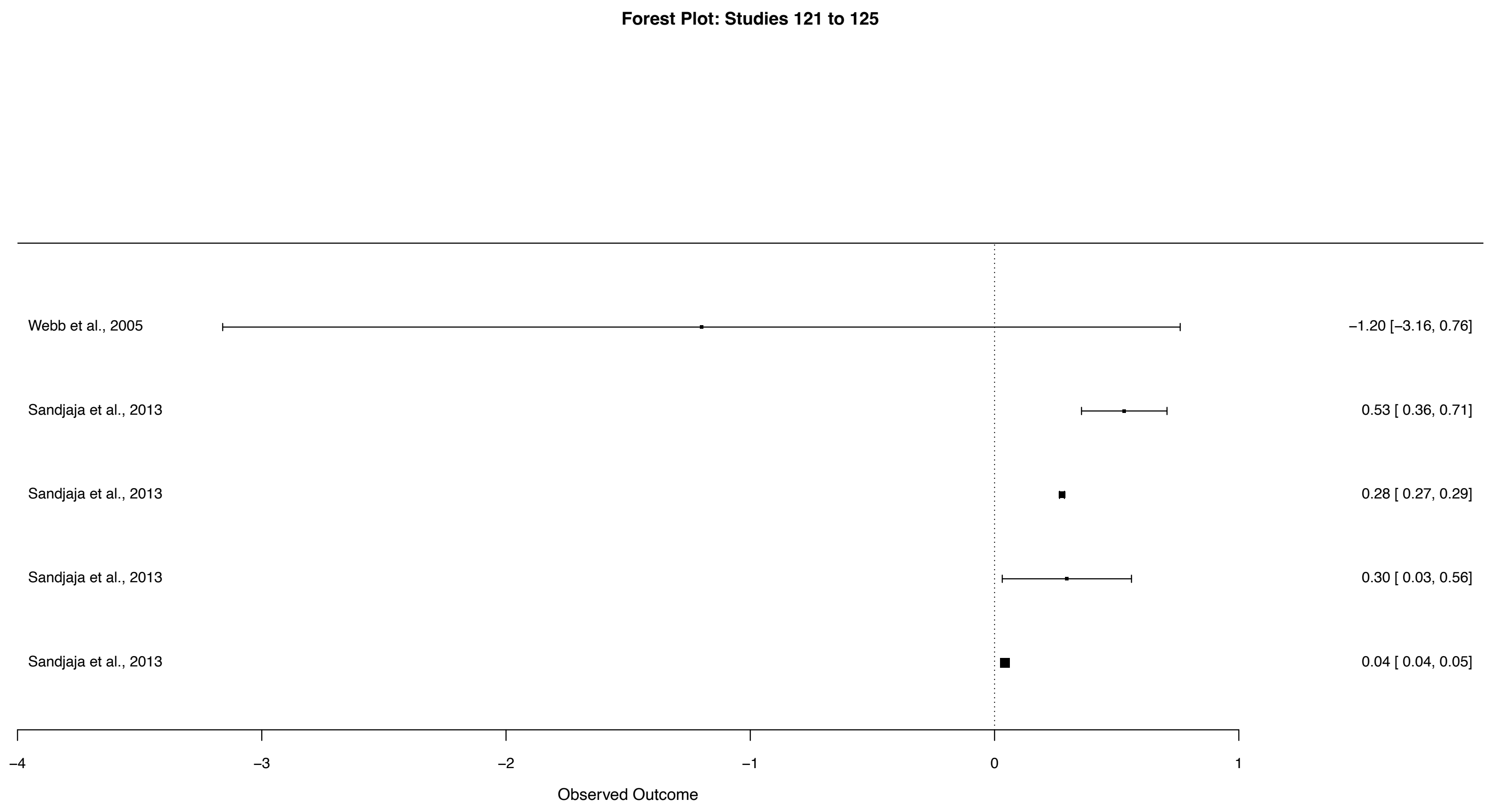

Forest Plot: Studies 121 to 125
Webb et al., 2005
−1.20 [−3.16, 0.76]
Sandjaja et al., 2013
0.53 [0.36, 0.71]
Sandjaja et al., 2013
0.28 [0.27, 0.29]
Sandjaja et al., 2013
0.30 [0.03, 0.56]
Sandjaja et al., 2013
0.04 [0.04, 0.05]
−4
−3
−2
−1
0
1
Observed Outcome

Figure S4. Funnel plot of the main model (including all studies).

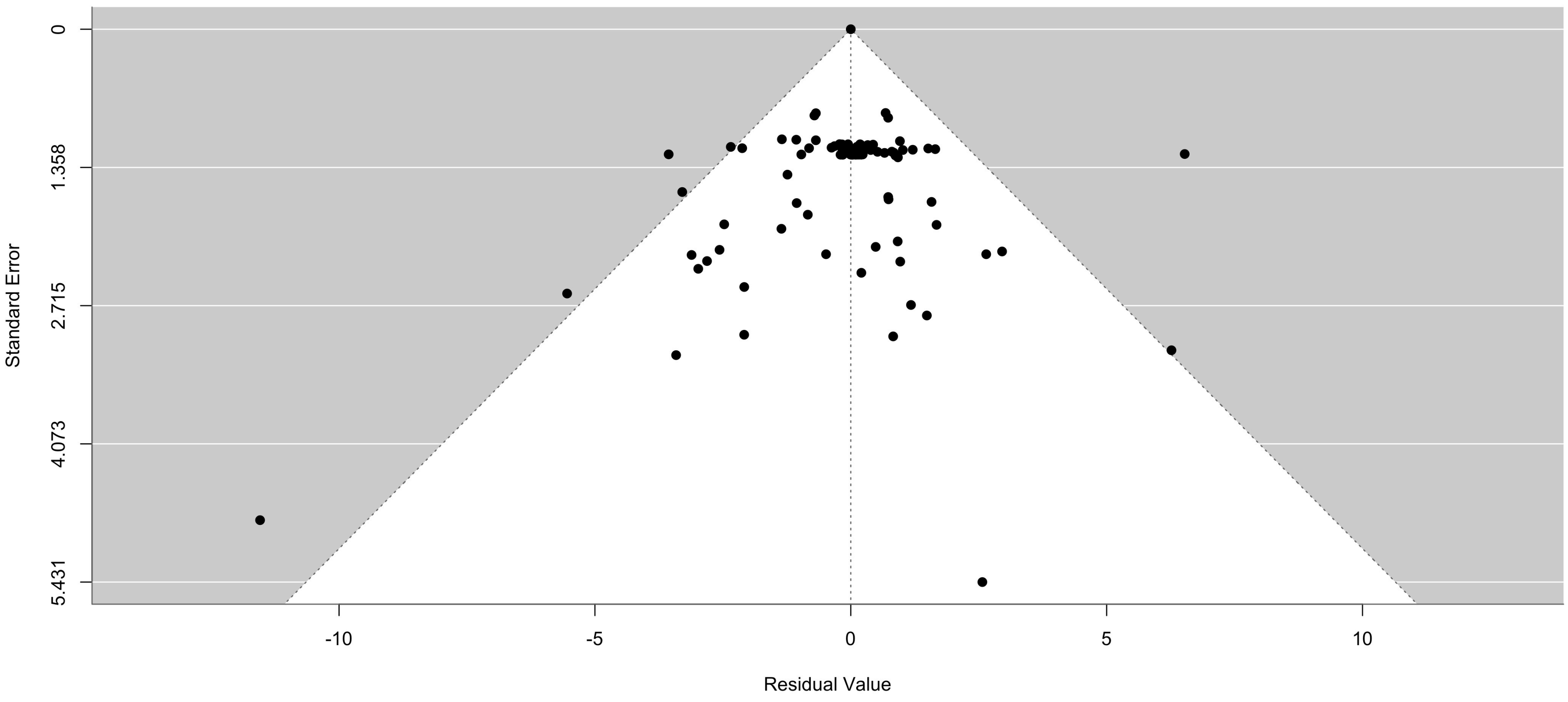

Sub-analyses of moderator effect on cognitive development in childhood stunting (3.2.1)

Table S10. Coefficients of all sub-domain models

Domain	Coefficient	Estimate	SE	Z value	p value	95% CI Lower	95% CI Upper	Significance
**Academic Skills**	intercept	-0.5881	1.2475	-0.4714	0.6373	-3.0332	1.8570	
**Academic Skills**	Age	0.4400	1.1931	0.3688	0.7123	-1.8985	2.7784	
**Academic Skills**	Region Asia	0.5611	0.6983	0.8035	0.4217	-0.8075	1.9297	
**Academic Skills**	Combined Factors (D, P)	-0.3363	1.5086	-0.2229	0.8236	-3.2930	2.6205	
**Academic Skills**	Combined Factors (H, P)	-0.4144	0.6226	-0.6657	0.5056	-1.6346	0.8058	
**Socioemotional Development**	intercept	-0.3310	0.4142	-0.7991	0.4242	-1.1428	0.4808	
**Socioemotional Development**	Region Asia	-0.0567	0.5229	-0.1084	0.9137	-1.0815	0.9682	
**Socioemotional Development**	Combined Factors (D, P)	0.3030	0.4445	0.6817	0.4954	-0.5681	1.1741	
**Socioemotional Development**	Combined Factors (D, S, H)	1.2570	2.8434	0.4421	0.6584	-4.3160	6.8299	
**Socioemotional Development**	**Combined Factors (D)**	**1.3877**	**0.4137**	**3.3540**	**0.0008**	**0.5768**	**2.1986**	***
**Cognition**	intercept	1.7231	1.8427	0.9351	0.3497	-1.8885	5.3346	
**Cognition**	Age	-1.8877	1.2856	-1.4684	0.1420	-4.4074	0.6319	
**Cognition**	Region America	0.8779	2.7350	0.3210	0.7482	-4.4826	6.2385	

**Cognition**	Region Asia	-1.9759	1.6917	-1.1680	0.2428	-5.2916	1.3398	
**Cognition**	Combined Factors (D, S, H, P)	-3.4951	2.3489	-1.4879	0.1368	-8.0989	1.1088	
**Cognition**	Combined Factors (D, S, H)	-0.4822	1.3504	-0.3571	0.7210	-3.1290	2.1646	
**Cognition**	Combined Factors (D, S)	-2.8244	2.8197	-1.0017	0.3165	-8.3508	2.7021	
**Cognition**	Combined Factors (D)	0.0638	1.7002	0.0375	0.9701	-3.2685	3.3962	
**Cognition**	Combined Factors (H)	-1.0505	1.4153	-0.7422	0.4579	-3.8244	1.7234	
**Cognition**	Combined Factors (S, H, P)	0.9405	2.0736	0.4536	0.6501	-3.1236	5.0046	
**Motor Skills**	intercept	0.1800	1.3595	0.1324	0.8947	-2.4845	2.8445	
**Motor Skills**	Age	-3.6828	1.9649	-1.8743	0.0609	-7.5339	0.1682	
**Motor Skills**	Region America	0.0291	1.9563	0.0149	0.9881	-3.8051	3.8634	
**Motor Skills**	Region Asia	-0.3600	1.9229	-0.1872	0.8515	-4.1288	3.4088	
**Intelligence**	**intercept**	**-1.3021**	**0.6508**	**-2.0007**	**0.0454**	**-2.5777**	**-0.0265**	*
**Intelligence**	Combined Factors (D, S, H)	-1.8797	2.0645	-0.9105	0.3626	-5.9261	2.1667	
**Intelligence**	Combined Factors (D)	1.5753	0.9055	1.7397	0.0819	-0.1994	3.3501	
**Oral Language**	intercept	1.0134	2.5390	0.3991	0.6898	-3.9630	5.9898	
**Oral Language**	Age	-1.9727	4.0028	-0.4928	0.6221	-9.8181	5.8727	
**Oral Language**	Region America	1.4475	3.6264	0.3992	0.6898	-5.6601	8.5550	
**Oral Language**	Combined Factors (D, S, H)	-1.1934	3.4647	-0.3444	0.7305	-7.9842	5.5974	

**Executive Function**	**intercept**	**0.0262**	**0.0084**	**3.1251**	**0.0018**	**0.0098**	**0.0427**	**
Memory	intercept	-1.6895	0.9717	-1.7387	0.0821	-3.5940	0.2150	

Table S11. Heterogeneity of all sub-domain models

Domain	QE	df	p value	Significance	tau2 (sigma21) Study ID	tau2 (sigma22) ES	I2_total
**Academic Skills**	**30398.3429**	**32**	**0**	***	**0.0000**	**2.0540**	**97.6338**
Socioemotional Development	1.1735	3	0.7594		0.0010	0.0000	0.0758
**Cognition**	**66.7390**	**13**	**3.18E-09**	***	**1.3227**	**0.6669**	**49.0039**
Motor Skills	5.3823	4	0.2503		0.9733	0.8724	40.3211
**Intelligence**	**1710.0757**	**4**	**0.0000**	***	**0.3855**	**0.0380**	**8.5851**
Oral Language	10.1595	5	0.0708		5.5550	0.0000	48.2193
**Executive Function**	**46.6038**	**31**	**0.0356**	*	**0.0000**	**0.0007**	**25.9846**
Memory	0.0124	1	0.9114		0.0000	0.0000	0.0000

Table S12. Moderators of all sub-domain models

Domain	QM	df	p value	Significance
Academic Skills	2.3507	5	0.7835	
**Socioemotional Development**	**15.7954**	**5**	**0.0000**	***

Cognition	11.3359	10	0.3273	
Motor Skills	5.0400	3	0.1689	
Intelligence	4.8209	2	0.0898	
Oral Language	1.3700	3	0.7126	
**Executive Function**	**9.7661**	**1**	**0.0018**	*
Memory	3.0232	1	0.0821	

Table S13. Publication bias test of all sub-domain models

Domain	Z value	p value	Significance
Academic Skills	-0.0023	0.9982	
Socioemotional Development	1.2048	0.2736	
Cognition	0.3231	0.7498	
Motor Skills	0.5255	0.6181	
Intelligence	0.7388	0.4932	
Oral Language	-1.1862	0.2743	
**Executive Function**	**-4.2023**	**0.0002**	***
